# Overexpression of the JmjC histone demethylase KDM5B in human carcinogenesis: involvement in the proliferation of cancer cells through the E2F/RB pathway

**DOI:** 10.1186/1476-4598-9-59

**Published:** 2010-03-13

**Authors:** Shinya Hayami, Masanori Yoshimatsu, Abhimanyu Veerakumarasivam, Motoko Unoki, Yukiko Iwai, Tatsuhiko Tsunoda, Helen I Field, John D Kelly, David E Neal, Hiroki Yamaue, Bruce AJ Ponder, Yusuke Nakamura, Ryuji Hamamoto

**Affiliations:** 1 Laboratory of Molecular Medicine, Human Genome Center, Institute of Medical Science, The University of Tokyo, 4-6-1 Shirokanedai, Minato-ku, Tokyo, 108-8639, Japan; 2 Second Department of Surgery, School of Medicine, Wakayama Medical University, 811-1, Kimiidera, Wakayama, 641-8510, Japan; 3 Department of Oncology, Cancer Research UK Cambridge Research Institute, University of Cambridge, Robinson Way, Cambridge, CB2 0RE, UK; 4 Medical Genetics Laboratory, Faculty of Medicine and Health Sciences, Universiti Putra Malaysia, 43400 Serdang, Selangor Darul Ehsan, Malaysia; 5 Laboratory for Biomarker, RIKEN, 4-6-1 Shirokanedai, Minato-ku, Tokyo, 108-8639, Japan; 6 Laboratory for Medical Informatics, RIKEN, 1-7-22 Suehirocho, Tsurumi-ku, Yokohama, Kanagawa, 230-0045, Japan; 7 Department of Genetics, University of Cambridge, Downing Street, Cambridge CB2 3EH, UK; 8 Division of Surgery & Interventional Science, UCL Medical School, University College London, 74 Huntley Street, London, WC1E 6AU, UK

## Abstract

**Background:**

Although an increasing number of histone demethylases have been identified and biochemically characterized, their biological functions largely remain uncharacterized, particularly in the context of human diseases such as cancer. We investigated the role of KDM5B, a JmjC histone demethylase, in human carcinogenesis. Quantitative RT-PCR and microarray analyses were used to examine the expression profiles of histone demethylases in clinical tissue samples. We also examined the functional effects of KDM5B on the growth of cancer cell lines treated with small interfering RNAs (siRNAs). Downstream genes and signal cascades induced by *KDM5B *expression were identified from Affymetrix Gene Chip experiments, and validated by real-time PCR and reporter assays. Cell cycle-dependent characteristics of KDM5B were identified by immunofluorescence and FACS.

**Results:**

Quantitative RT-PCR analysis confirmed that expression levels of *KDM5B *are significantly higher in human bladder cancer tissues than in their corresponding non-neoplastic bladder tissues (*P *< 0.0001). The expression profile analysis of clinical tissues also revealed up-regulation of *KDM5B *in various kinds of malignancies. Transfection of KDM5B-specific siRNA into various bladder and lung cancer cell lines significantly suppressed the proliferation of cancer cells and increased the number of cells in sub-G_1 _phase. Microarray expression analysis indicated that E2F1 and E2F2 are downstream genes in the KDM5B pathway.

**Conclusions:**

Inhibition of KDM5B may affect apoptosis and reduce growth of cancer cells. Further studies will explore the pan-cancer therapeutic potential of KDM5B inhibition.

## Background

Histone methylation plays an important dynamic role in regulating chromatin structure. Precise coordination and organization of open and closed chromatins are crucial for normal cellular processes such as DNA replication, repair, recombination and transcription. Until recently, histone methylation was considered to be a static modification, but the identification of histone demethylases has revealed that this modification is dynamically regulated [1,2]. Histone demethylases regulate not only the modification itself but also its extended function, by antagonizing the binding of effector proteins to modified chromatin. This is exemplified by JHDM3A/JMJD2A, which displaces HP1 from chromatin by demethylating the H3K9 methylation and thereby preventing the spread of H3K9 methylation to the surrounding chromatin by HP1 [3,4]. A highly-conserved family of proteins containing the JmjC domain was recently characterized to possess a histone demethylase activity [5]. Despite a large body of information for the prominent role of histone demethylases in transcriptional regulation, their physiological function, and their involvement in human disease is still not well-understood.

We previously reported that SMYD3, a histone methyltransferase, stimulates cell proliferation through its methyltransferase activity and plays a crucial role in human carcinogenesis [6-10]. Although dysfunction of histone methylation status was indicated to contribute to human carcinogenesis [11-13], the relationship between abnormal histone demethylation and human carcinogenesis is still largely unclear.

In order to find demethylases that contribute to human carcinogenesis, we examined the expression profiles of several proteins containing a JmjC histone demethylase domain in clinical tissues and found that expression levels of KDM5B were significantly up-regulated, compared with their corresponding normal tissues, in many types of cancer.

KDM5B, also named JARID1B or PLU-1, is one of the four JARID family members [14,15], and contains domains common to transcriptional regulators such as a JmjN domain, a Bright/Arid domain, a C5H2C zinc finger motif, and several PHD domains in addition to a JmjC domain. All four members of the JARID family possess the H3K4 demethylase activity [16-20]. Each member might participate in different biological processes through recruitment to different chromosomal regions and differing enzymatic activities [5]. Here we demonstrate a novel function of KDM5B in human carcinogenesis and show that it is related to the cell cycle through regulation of E2F expression and cell growth.

## Results

### KDM5B expression is up-regulated in clinical cancer tissues

**Figure 1:**
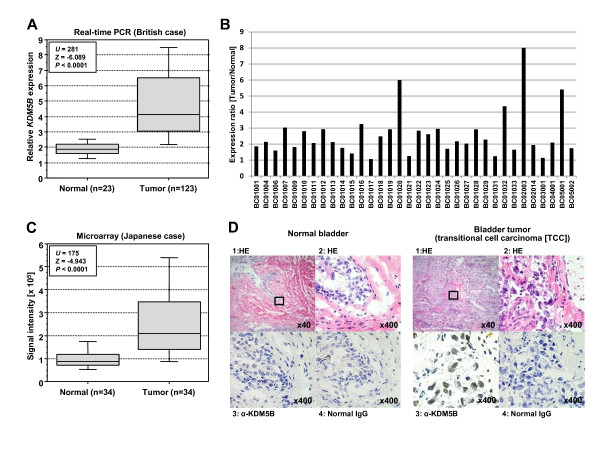
**Elevated KDM5B expression in bladder cancer in British and Japanese patients**. (A) *KDM5B *gene expression in normal and tumor bladder tissues in British cases. Expression levels of *KDM5B *were analyzed by quantitative real-time PCR, and the result is shown by box-whisker plot (median 50% boxed). Relative mRNA expression shows the value normalized by *GAPDH *and *SDH *expressions. Mann-Whitney's *U-*test was used for statistical analysis. (B) Expression ratio between bladder normal and tumor tissues in Japanese patients. Signal intensity for each sample was analyzed by cDNA microarray, and the expression ratio is the signal intensity in tumor divided by that in normal (1 is normal). (C) Comparison of *KDM5B *expression between normal and tumor bladder tissues in Japanese patients. Signal intensity of each sample was analyzed by cDNA microarray, and the result is shown by box-whisker plot (median 50% boxed). Mann-Whitney's *U-*test was used for statistical analysis. (D) Immunohistochemical staining of KDM5B in bladder tissues. Nonimmunized mouse IgG was used as a substitute for the primary antibody to eliminate the possibility of false-positive responses from non-specific binding of IgG or from the secondary antibody. Counterstaining was done with hematoxylin and eosin. Original magnification, ×40 and ×400.

We first examined expression levels of five jumonji histone demethylase genes included in JARID family, *KDM5A (JARID1A)*, *KDM5B (JARID1B)*, *KDM5C (JARID1C)*, *KDM5D (JARID1D) *and *JARID2*, in a small subset of clinical bladder cancer samples and found a significant difference in expression levels between normal and cancer cells only for the KDM5B gene (data not shown). Therefore, we analyzed 123 bladder cancer samples and 23 normal control samples (British) and confirmed significant elevation of *KDM5B *expression in tumor cells compared with in normal cells (*P *< 0.0001, Mann-Whitney's *U*-test) (Figure 1A and Additional file 1). No significant difference was observed in expression levels among different grades and stages (Table 1 and Additional file 1). This suggests that *KDM5B *expression was up-regulated in an early stage of bladder carcinogenesis, and remained high in the advanced stages of the disease. Subclassification of tumors according to gender, smoking history, metastasis status, and recurrence status identified no significant difference in the expression levels of *KDM5B *(Table 1). We then analyzed the expression patterns of *KDM5B *in a number of clinical samples derived from Japanese bladder cancer subjects examined by cDNA microarray (Figure 1B and 1C), and confirmed its significant overexpression (*P *< 0.0001, Mann-Whitney's *U*-test).

**Table 1:** Statistical analysis of *KDM5B *expression levels in clinical bladder tissues

		*KDM5B *expression		
		
Factor	n	Mean	SD	95%CI
**Normal (Control)**	23	1.935	0.549	1.698 - 2.173
				
**Tumor (Total)**	123	5.018	3.322	4.425 - 5.611
Gender				
Male	90	5.032	3.378	4.324 - 5.739
Female	31	4.477	2.243	3.654 - 5.299
Smoke				
No	27	4.702	2.246	3.813 - 5.590
Yes	49	5.549	4.050	4.386 - 6.712
Grade				
G1	12	5.035	2.654	3.349 - 6.720
G2	62	5.740	3.982	4.728 - 6.751
G3	48	4.079	2.196	3.441 - 4.717
Metastasis				
Negative	96	4.988	3.547	4.269 - 5.706
Positive	27	5.125	2.408	4.173 - 6.078
Recurrence				
No	27	5.606	4.735	3.733 - 7.480
Yes	50	4.988	2.540	4.266 - 5.710
Died	8	5.948	3.280	3.205 - 8.690

**Figure 2:**
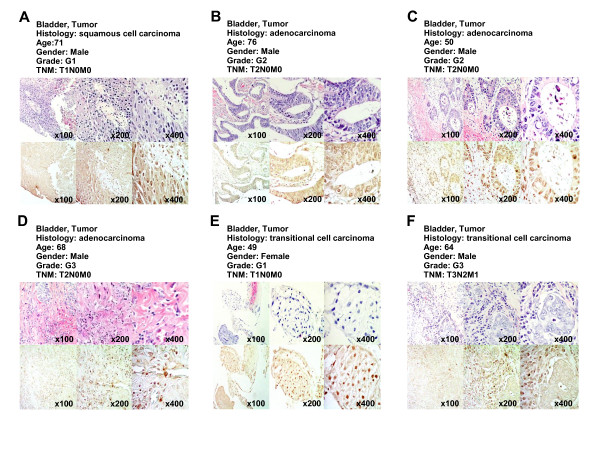
**Tissue microarray images of bladder tumors stained by standard immunohistochemistry for protein expression of KDM5B**. Clinical information for each section is represented above histological pictures. Counterstaining was done with hematoxylin and eosin. Original magnification, ×100, ×200 and ×400.

To evaluate protein expression levels of KDM5B in bladder tissues, we performed immunohistochemical analysis using anti-KDM5B specific antibody (Figure 1D). We observed strong KDM5B staining mainly in the nucleus of malignant cells, but no significant staining in non-neoplastic tissues. To further validate this result, we conducted tissue microarray experiments using 29 bladder tissue sections (Figure 2 and Additional file 2), and observed strong staining in 6 cases, and weak or moderate staining was observed in 13 cases. Moreover, no significant relationship between KDM5B protein expression levels and clinicopathologic characteristics was detected, consistent with our real-time PCR results.

**Figure 3:**
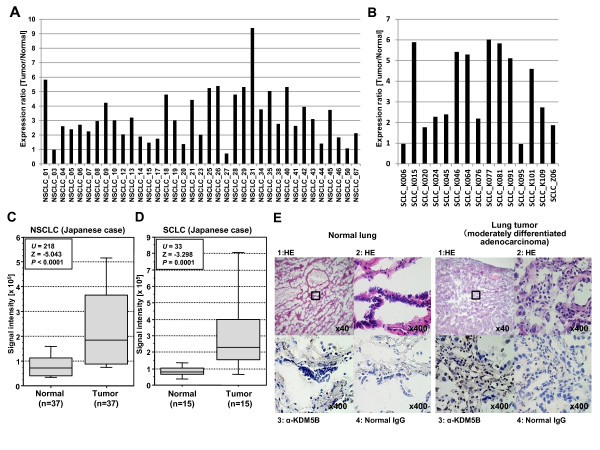
**Elevated KDM5B expression in lung cancer**. (A and B) Expression ratio between normal lung and tumor (non-small cell lung cancer [NSCLC] in A and small cell lung cancer [SCLC] in B) lung tissues in Japanese patients. Signal intensity for each sample was analyzed by cDNA microarray, and the expression ratio is the signal intensity in tumor divided by that in normal (1 is normal). (C and D) Comparison of *KDM5B *expression between normal and tumor (NSCLC and SCLC) lung tissues. Signal intensity of each sample was analyzed by cDNA microarray, and the result is shown by box-whisker plot (median 50% boxed). Mann-Whitney's *U-*test was used for the statistical analysis. (E) Immunohistochemical staining of KDM5B in lung tissues. Nonimmunized mouse IgG was used as a substitute for the primary antibody to verify the possibility of false-positive responses from non-specific binding of IgG or from the secondary antibody. Counterstaining was done with hematoxylin and eosin. Original magnification, ×40 and ×400.

**Figure 4:**
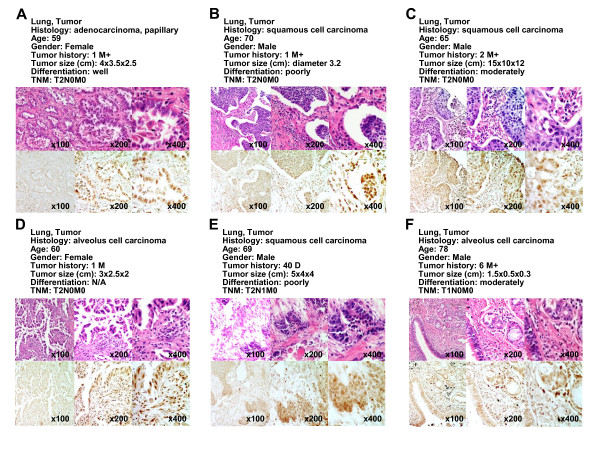
**Tissue microarray images of lung tumors stained by standard immunohistochemistry for protein expression of KDM5B**. Clinical information for each section is represented above histological pictures. Counterstaining was done with hematoxylin and eosin. Original magnification, ×100, ×200 and ×400.

In addition to bladder tissues, we measured expression levels of KDM5B in lung tissues (Figure 3 and 4). cDNA microarray experiments showed that *KDM5B *expression was also highly elevated in lung tumor tissues compared with corresponding non-neoplastic tissues (Figure 3A-D). Importantly, elevated *KDM5B *expression was observed in both non-small cell lung cancers and small cell lung cancers, indicating that *KDM5B *overexpression is involved widely in lung carcinogenesis. We then examined KDM5B protein expression levels in lung tissue by immunohistochemistry (Figure 3E). We observed strong KDM5B staining in cancer tissues and no significant staining in non-neoplastic tissues. To evaluate protein expression levels of KDM5B in various types of lung tumor tissues, we conducted tissue microarray experiments (Figure 4 and Additional file 3). Among 62 tumor tissue sections examined, we observed strong staining in 16 cases, and weak or moderate staining in 28 cases. In addition, we examined microarray expression analysis data of a large number of clinical samples derived from Japanese subjects and found that *KDM5B *expression was also significantly up-regulated in acute myelogenous leukemia (AML), breast cancer, chronic myelogenous leukemia (CML), cervical cancer and renal cell carcinoma (RCC) compared with corresponding non-neoplastic tissues, indicating its possible involvement in many types of human cancer (see Additional files 4 and 5).

### Growth regulation of cancer cells by KDM5B

**Figure 5:**
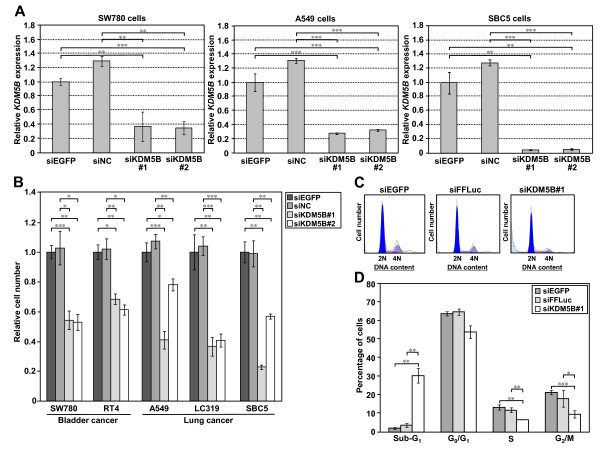
**Involvement of KDM5B in the growth of bladder and lung cancer cells**. (A) Quantitative real-time PCR showing suppression of endogenous expression of *KDM5B *by two KDM5B-specific siRNAs (siKDM5B#1 and #2) in SW780, A549 and SBC5 cells. siEGFP and siNC were used as controls. mRNA expression levels were normalized by *GAPDH *and *SDH *expressions, and values are relative to siEGFP (siEGFP = 1). Results are the mean ± SD of three independent experiments. *P*-values were calculated using Student's *t*-test (**, *P *< 0.01; ***, *P *< 0.001). (B) Effect of *KDM5B *siRNA knockdown on the viability of bladder cancer cell lines (RT4 and SW780) and lung cancer cell lines (A549, LC319 and SBC5). Relative cell number shows the value normalized to siEGFP-treated cells. Results are the mean ± SD in three independent experiments. *P*-values were calculated using Student's *t*-test (*, *P *< 0.05; **, *P *< 0.01; ***, *P *< 0.001). (C) DNA content of SBC5 cells analyzed by FACS 72 hours after the treatment with control siRNAs and siKDM5B#1. We show representative histograms for each experiment. (D) Numerical analysis of the FACS results in C, classifying cells by cell cycle status. The proportion of cancer cells in sub-G_1 _phase is significantly high after treatment with siKDM5B compared to control siRNAs-treated cancer cells. Results are the mean ± SD in three independent experiments. *P*-values were calculated using Student's *t*-test (*, *P *< 0.05; **, *P *< 0.01; ***, *P *< 0.001).

To investigate the role of KDM5B in human carcinogenesis, we performed a knockdown experiment using two independent siRNAs against KDM5B (siKDM5B#1 and #2) and two control siRNAs (siEGFP and siNC). We transfected these siRNAs into SW780, A549 and SBC5 cells in which *KDM5B *was highly expressed (see Additional file 6A). Expression levels of *KDM5B *in the cells transfected with two independent siRNAs targeting KDM5B were significantly suppressed, compared to those transfected with control siRNAs (Figure 5A). Using the same siRNAs, we performed cell growth assays and found significant growth suppression by two independent siRNAs targeting KDM5B for two bladder cancer cell lines (SW780 and RT4) and three lung cancer cell lines (A549, LC319 and SBC5) while no effect was observed when we used control siRNAs (Figure 5B).

To further assess the mechanism of growth suppression induced by the siRNA, we analyzed the cell cycle status of cancer cells after treatment with siRNAs using flow cytometry (Figure 5C). The proportion of cancer cells at the sub-G_1 _phase was significantly higher in the cells treated with siKDM5B than those treated with control siRNAs (*P *= 0.0055 [siEGFP, siKDM5B] and *P *= 0.0042 [siFFLuc, siKDM5B], respectively, Figure 5D). As increasing the number of cells at sub-G_1 _phase has been considered as a marker of apoptosis [21], it is possible that apoptosis can be induced by knockdown of KDM5B expression. We also performed BrdU and 7-AAD staining analysis, and the data are concordant with PI-staining FACS analysis (see Additional file 6B).

### Identification of the downstream genes by microarray expression analysis

We then performed microarray expression analysis to identify signal pathways of downstream of KDM5B. In order to clarify early responding genes after knockdown of *KDM5B*, we isolated total RNA from SW780 and A549 cells, 24 hours after treatment with siKDM5B. The expression profile analysis of these cells using Affymetrix HG-U133 Plus 2.0 Array in comparison with those treated with control siRNAs (siEGFP and siFFLuc) identified a set of genes significantly up/down-regulated. We further performed a signal pathway analysis, referring to the Gene Ontology database (Methods and Additional file 7), and found that KDM5B could be closely linked with the process of cell cycle regulation. Interestingly, we observed significant down-regulation of E2F1 and E2F2 by treatment with siKDM5B (*P *< 0.0001 for both) (see also Additional file 8). As the E2F/RB pathway is a key regulator of the cell cycle, we analyzed the functional relationship between *KDM5B *expression and this pathway.

**Figure 6:**
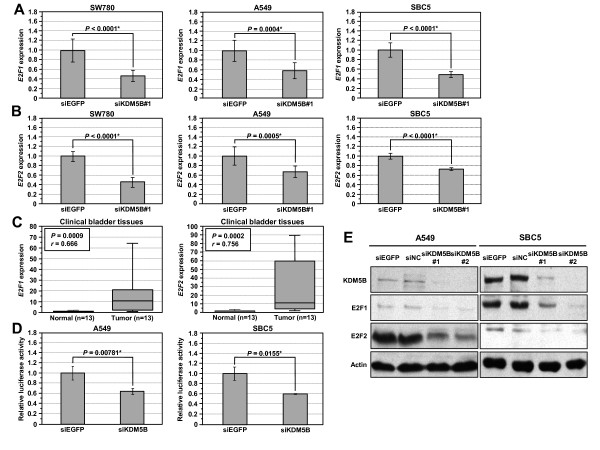
**Confirmation of E2F1 and E2F2 as downstream genes of KDM5B**. (A and B) Expression levels of *E2F1 *(A) and *E2F2 *(B) in SW780, A549 and SBC5 cells were analyzed by real-time PCR after treatment with siRNAs targeting *KDM5B *(siKDM5B) and *EGFP *(control; siEGFP = 1). Relative mRNA expression is an arbitrary value, but for each cell population, expression was normalized to *GAPDH *and *SDH *expressions. Statistical analysis was based on nine independent experiments. *P*-values were calculated using Student's *t*-test. (C) *E2F1 *and *E2F2 *expressions as measured by real-time PCR were significantly up-regulated in bladder tumor tissues compared to non-neoplastic bladder tissues, in proportion to that of *KDM5B *expression in tumor tissue. We analyzed 13 cancer and normal tissues, and Spearman's rank correlation coefficient was used for the statistical analysis. (D) Knockdown of *KDM5B *expression repressed the transcriptional activity of E2F. Results are the mean ± SD in three independent experiments. *P*-values were calculated using Student's *t*-test. (E) Validation of KDM5B and E2F1 expressions at the protein level. Lysates from A549 and SBC5 cells after both siKDM5B#1 and siKDM5B#2 treatments were immunoblotted with anti-KDM5B, E2F1, E2F2 and actin antibodies. Expression of actin served as an internal control.

We confirmed the down-regulation of E2F1 and E2F2 expression in three different cancer cell lines, SW780, A549 and SBC5 treated with siRNAs, by quantitative real-time PCR (Figure 6A and 6B). Moreover, we also found higher expression levels of both E2F1 and E2F2 in clinical tumor tissues where *KDM5B *was overexpressed, than in non-neoplastic tissues (*P *= 0.0009 and *P *= 0.0002, respectively). The data indicate that both E2F1 and E2F2 could be highly expressed in tumor tissues correlating with elevated expression of *KDM5B *(Spearman's rank correlation coefficient: *r *= 0.666 [E2F1] and *r *= 0.756 [E2F2], respectively) (Figure 6C).

To validate the transcriptional regulation of E2F by KDM5B in more detail, we performed luciferase reporter assays using an E2F-responsive luciferase construct (Figure 6D). We transfected the construct into cancer cell lines after treatment with siEGFP or siKDM5B. The E2F-driven transcriptional activity was significantly suppressed after treatment with siKDM5B in both A549 and SBC5 cells. Furthermore, we also confirmed suppression of both E2F1 and E2F2 expressions in A549 and SBC5 cells at the protein level after treatment with two independent siRNAs targeting KDM5B (Figure 6E). These results reveal that the transcriptional activity regulated by E2F transcription factors can be suppressed after knockdown of *KDM5B*, and this disruption of this pathway may be responsible for the cell cycle alterations which we have observed.

## Discussion

Histone modifications of chromatin, including methylation, acetylation, phosphorylation and ubiquitination, play a critical role in creating transcriptional activation and repression patterns, through the regulation of chromatin structure. KDM5B belongs to the lysine demethylase family, which specifically removes the methyl group of histone H3 lysine 4 [20]. In this study, we demonstrated the significant up-regulation of KDM5B in bladder and lung cancers as well as various other cancer types, using quantitative RT-PCR, immunohistochemistry, and microarray-based gene expression profiles. Consistently with reports from other groups [20,22], we showed that KDM5B expression is dysregulated in a great majority of human tumors. We previously reported the copy number gain at a region of chromosome 1q32.1 (200963156-201045186), where the *KDM5B *gene is located, in 17 of 98 bladder tumors (British cases) [23]. We found that the clone RP11-203F10 (200861805-201051225) used for this tile-path array analysis included an entire *KDM5B *gene. Although most of the cases were considered to be transactivated by other mechanisms, including epigenetic changes or activation of factors regulating the KDM5B transcription, the copy number gain in nearly 20% of the cases indicated some significances of this copy number gain in bladder carcinogenesis.

KDM5B expression is very low in normal tissues other than adult testis [24], and we observed no significant KDM5B staining in vital organs by immunohistochemical analysis (Figure 1D, 3E and Additional file 9). Therefore, aberrant overexpression of KDM5B in any tumor, compared to corresponding non-neoplastic tissues, make it an ideal molecular target with potential for cancer detection and as a therapeutic target. Already, synthetic inhibitors of classical HDACs have been widely used as tools in epigenetic studies, and many have shown growth-suppressive effects in cancer cells *in vitro *and have been used in early phase clinical trials [25,26]. In addition, some histone methyltransferase and demethylase inhibitors have recently been reported [27-29]. Further functional studies of KDM5B will provide useful information for development of demethylase inhibitors that might show a great promise as a new type of molecular targeted-cancer drugs, as well as HDAC inhibitors.

We demonstrated that E2F1 and E2F2 are candidate downstream modulators regulated by KDM5B. A luciferase reporter assay, combined with siRNA treatment, yielded indirect evidence supporting a molecular interaction between KDM5B and E2F elements. The E2F transcription factors are downstream effectors of the retinoblastoma (RB) protein pathway and are involved in many aspects of fundamental cell cycle control [30-33]. Binding sites for E2F factors have been identified in a large number of genes that control cell cycle and DNA synthesis, including cdk2 and 4, cyclin A, D and E, DNA polymerase, ribonucleotide reductase, UHRF1 and PCNA [34,35]. Importantly, mutations in the RB-E2F cascade are found in a wide range of tumor types [36,37]. Most of these alterations affect RB or upstream regulators of E2F transcriptional factors, and there is growing evidence that dysregulation of the E2F family itself is crucially involved in carcinogenesis. Indeed, in ovarian cancer, the proliferation-promoting E2F1 and E2F2 transcription factors were overexpressed, compared with healthy control tissues [38]. Their dysregulation has been proposed as a prognostic indicator for various tumors [39-43]. Overexpression of a proliferation-promoting E2F transcription factor is argued to contribute a significant growth advantage to tumors especially those with poor prognosis. In the present study, we demonstrated significantly higher expression of both E2F1 and E2F2 in bladder tumor tissues than in non-neoplastic tissues, which are probably due to aberrant transcriptional regulation of KDM5B. Detailed pathway analysis on the basis of Gene Ontology revealed the involvement of KDM5B in several cell cycle processes (see also Additional file 7).

According to our microarray data, a number of other genes could be up-regulated by KDM5B. One of KDM5B's main functions was considered to be transcriptional repression through its demethylase activity because H3K4 methylation is a marker for euchromatin [20]. From our microarray data, we propose three possible mechanisms whereby KDM5B can activate the transcription of its downstream genes. (i) Transcription is indirectly activated through transcription factors that are directly regulated by KDM5B; (ii) KDM5B transactivates expression of downstream candidates through protein-protein interaction. For example, KDM5B associates with the androgen receptor and enhances its transcriptional activity [22]. KDM5B might both up- and down-regulate gene expressions, depending on its binding partners. (iii) KDM5B demethylates unknown substrates. Similarly, LSD1 was first reported to be a H3K4 specific demethylase [1], and later found to demethylate histone H3 lysine 9 and p53 [44,45]. Interestingly, in this study, we found that KDM5B was localized in the cytoplasm at some cell-cycle phases (see Additional files 10 and 11), raising the possibility that it might demethylate unknown substrates in the cytoplasm and then affect cell cycle progression. Furthermore, Xiang *et al *has shown that there may be a correlation between *KDM5B *expression and the stage of prostate cancer [22] and Yamane *et al *reported that KDM5B knockdown increased G_1 _phase of MCF7 cells [20]. While these are some discrepancies between our current result and the previous reports, these differences may reflect the different KDM5B roles in different tissues. However, our results using several cancer cell lines strongly support the possible involvement of KDM5B in the growth of cancer cells.

## Conclusions

The present study identified high expression of *KDM5B*, a JmjC histone demethylase, in the majority of bladder tumor tissues analyzed by real-time PCR. Microarray data indicated significantly higher levels of *KDM5B *expression in many types of tumor tissues compared to corresponding non-neoplastic tissues. We showed that reduction of *KDM5B *expression resulted in suppression of cell growth of cancer cells, through co-regulation of the E2F/RB1 cell cycle regulation pathway, and possibly the promotion of apoptosis of cells remaining in sub-G_1 _phase. As significant high expression of KDM5B was only observed in cancer cells, and its knockdown suppressed the growth of cancer cells, it may be an ideal druggable molecular target. Further functional analyses of this protein could contribute to development of novel therapeutic strategies for bladder and other carcinomas.

## Materials and methods

### Tissue samples and RNA preparation

123 surgical specimens of primary urothelial carcinoma were collected, either at cystectomy or transurethral resection of bladder tumor (TUR-Bt), and snap-frozen in liquid nitrogen. 23 normal bladder tissues were collected from areas of macroscopically-normal regions in patients with no evidence of malignancy. Use of tissues for this study was approved by Cambridgeshire Local Research Ethics Committee (Ref 03/018). A total of thirty 30-μm sections were homogenized for RNA extraction and two 7-μm 'sandwich' sections adjacent to the tissue used for RNA extraction were sectioned, stained and assessed for cellularity and tumor grade by an independent consultant urohistopathologist. Additionally, the sections were graded according to the degree of inflammatory cell infiltration (low, moderate and significant). Samples showing significant inflammatory cell infiltration were excluded [46].

Total RNA was extracted using TRI Reagent™ (Sigma, Dorset, UK), following the manufacturer's protocol. RNeasy Mini Kit™ (QIAGEN, Crawley, UK), including a DNase step, was used to optimize RNA purity. Agilent 2100™ total RNA bioanalysis was performed. 1 μl of resuspended RNA from each sample was applied to an RNA 6000 Nano LabChip™ and processed according to the manufacturer's instructions. All chips and reagents were sourced from Agilent Technologies™ (West Lothian, UK).

### Reverse transcription

Total RNA concentrations were determined using the NanoDrop™ ND1000 spectrophotometer (Nyxor Biotech, Paris, France). 1 μg of total RNA was reversely transcribed with 2 μg of random hexamers (Amersham) and Superscript III reverse transcriptase (Invitrogen, Paisley, UK) in 20 μl reactions according to the manufacturer's instructions. cDNA was then diluted 1:100 with PCR grade water and stored at -20°C.

### Laser capture microdissection

Tissue for laser capture microdissection was collected prospectively following the procedure outlined above. Five sequential sections of 7-μm thickness were cut from each tissue and stained using HistoGene™ staining solution (Arcturus, California, USA) following the manufacturer's protocol. Slides were then immediately transferred for microdissection using a PixCell II laser capture microscope™ (Arcturus, California, USA). This technique employs a low-power infrared laser to melt a thermoplastic film over the cells of interest, to which the cells become attached.

Approximately 10,000 cells were microdissected from both stromal and epithelial/tumor compartments in each tissue. RNA was extracted using an RNeasy Micro Kit™ (QIAGEN, Crawley, UK). Areas of cancer or stroma containing significant inflammatory areas of tumor or stroma containing significant inflammatory cell infiltration were avoided to prevent contamination.

Total RNA was reversely transcribed and qRT-PCR was performed as shown above. Given the low yield of RNA from such small samples, NanoDrop™ quantification was not performed, but correction for the endogenous 18S CT value was used as an accurate measure of the amount of intact starting RNA. Transcript analysis was performed for the *KDM5B *genes.

To validate the accuracy of microdissection, qRT-PCR using primers and probes for Vimentin and Uroplakin were performed according to the manufacturer's instructions (Assays on demand, Applied Biosystems, Warrington, UK). Vimentin is primarily expressed in mesenchymal tissue, and was used as a stromal marker. Uroplakin, a marker of urothelial differentiation, is preserved in up to 90% of epithelially-derived cells [47].

### Cell culture

All cell lines were grown in monolayers in appropriate media: Dulbecco's modified Eagle's medium (D-MEM) for EJ28 bladder cancer cells and RERF-LC-AI non-small cell lung cancer cells; Eagle's minimal essential medium (E-MEM) for 253J, 253J-BV, HT1197, HT1376, J82, SCaBER, UMUC3 bladder cancer cells and SBC5 small cell lung cancer cells; Leibovitz's L-15 for SW780 cells; McCoy's 5A medium for RT4 and T24 bladder cancer cells; RPMI1640 medium for 5637 bladder cancer cells and A549, H2170 and LC319 non-small cell lung cancer cells supplemented with 10% fetal bovine serum and 1% antibiotic/antimycotic solution (Sigma). All cells were maintained at 37°C in humid air with 5% CO_2 _condition (5637, 253J, 253J-BV, EJ28, HT1197, HT1376, J82, RT4, SCaBER, T24, UMUC3, A549, H2170, LC319, RERF-LC-AI and SBC5) or without CO_2 _(SW780). Cells were transfected with FuGENE6™ (Roche Applied Science, Basel, Switzerland) according to manufacturer's protocols.

### Expression profiling in cancers using cDNA microarrays

We established a genome-wide cDNA microarray with 36,864 cDNAs or ESTs selected from the UniGene database of the National Center for Biotechnology Information (NCBI). This microarray system was constructed essentially as described previously [48-50]. Briefly, the cDNAs were amplified by RT-PCR using poly (A)^+ ^RNAs isolated from various human organs as templates; the lengths of the amplicons ranged from 200 to 1,100 bp, without any repetitive or poly (A) sequences. Many types of tumors and corresponding non- neoplastic tissues were prepared in 8-μm sections, as described previously [49]. A total of 30,000-40,000 cancer or noncancerous cells were collected selectively using the EZ cut system (SL Microtest GmbH, Germany) according to the manufacturer's protocol. Extraction of total RNA, T7-based amplification, and labeling of probes were performed as described previously [49]. 2.5-μg aliquots of twice-amplified RNA (aRNA) from each cancerous and noncancerous tissue were then labeled, respectively, with Cy3-dCTP or Cy5-dCTP. Detailed expression profiling data of bladder and lung cancers, shown in this study, were based on the data reported previously by Drs. Ryo Takata and Takefumi Kikuchi, respectively [48,51].

### Quantitative real-time PCR

As described above, we obtained 123 bladder cancer tissues and 23 normal bladder tissues in Cambridge Addenbrooke's Hospital. For quantitative RT-PCR reactions, specific primers for all human *GAPDH *(housekeeping gene), *SDH *(housekeeping gene) and *KDM5B *were designed (primer sequences in Additional file 12). PCR was performed using the ABI prism 7700 Sequence Detection System (Applied Biosystems, Warrington, UK) following the manufacturer's protocol. 50% SYBR GREEN universal PCR Master Mix without UNG (Applied Biosystems, Warrington, UK), 50 nM each of the forward and reverse primers and 2 μl of reversely-transcribed cDNA were applied. Amplification conditions were 5 min at 95°C and then 45 cycles each consisting of 10 sec at 95°C, 1 min at 55°C and 10 sec at 72°C. Then, reactions were heated for 15 sec at 95°C, 1 min at 65°C to draw the melting curve, and cooled to 50°C for 10 sec. Reaction conditions for target gene amplification were as described above and the equivalent of 5 ng of reverse transcribed RNA was used in each reaction. mRNA levels were normalized to *GAPDH *and *SDH *expressions.

### Immunohistochemical staining

Sections of human bladder and lung cancer were stained by VECTASTAIN^® ^ABC KIT (VECTOR LABORATORIES, CA, USA). Briefly, endogenous peroxidase activity of xylene-deparaffinized and dehydrated sections was inhibited by treatment with 0.3% H_2_O_2_/methanol. Non-specific binding was blocked by incubating sections with 3% BSA in a humidified chamber for 30 min at ambient temperature followed by overnight incubation at 4°C with a 1:100 dilution of mouse monoclonal anti-JARID1B (clone 1G10, Abnova) antibody. The sections were washed twice with PBS (-), incubated with 5 μg/μl goat anti-mouse biotinylated IgG in PBS (-) containing 1% BSA for 30 min at ambient temperature, and then incubated with ABC reagent for 30 min. Specific immunostaining was visualized by 3,3'-diaminobenzidine. Slides were dehydrated through graded alcohol to xylene washing and mounted on cover slips. Hematoxylin was used for nuclear counterstaining. Bladder and lung tumor tissue microarray slides were purchased from BioChain Institute (Hayward, CA, USA).

### Western blotting

Total protein extracts were prepared from the cells in RIPA-like buffer. Total protein (50 μg) was transferred to nitrocellulose membrane. The membrane was proved with anti-JARID1B (clone 1G10, H00010765-M02, Abnova or HPA027179, Atlas Antibodies AB), anti-E2F1 antibody (KH95, Santa Cruz Biotechnology) and anti-E2F2 antibody (L-20, Santa Cruz Biotechnology). Anti-Actin (I-19, Santa Cruz Biotechnology) was used as loading control.

### Transfection with small interfering RNAs

The small interfering RNA *(*siRNA) oligonucleotide duplexes were purchased from SIGMA Genosys for targeting the human *KDM5B *transcript. siEGFP, siFFLuc and siNegative control (siNC), which is a mixture of three different oligonucleotide duplexes, were used as control siRNAs. The siRNA sequences are described in Additional file 13. siRNA duplexes (100 nM final concentration) were transfected to bladder and lung cancer cell lines with lipofectamine 2000 (Invitrogen) for 72 hours, and cell viability was examined using Cell Counting Kit 8 (DOJINDO Laboratories).

### Flow cytometry assays (FACS)

To examine the effect of KDM5B expression on the cell cycle progression, SBC5 cells were treated with siKDM5B or control siRNAs (siEGFP and siFFLuc), and cultured in a CO_2 _incubator at 37°C for 72 hours. 1 × 10^5 ^cells were collected by trypsinization, and stained with propidium iodide (PI) following the manufacturer's instructions (Cayman Chemical). Cells were analyzed by FACScan (BECKMAN COULTER) with MultiCycle for Windows software (BECKMAN COULTER) for detailed cell cycle status. The percentages of cells in G_0_/G_1_, S and G_2_/M phases as well as those in any sub-G_1 _population were determined from at least 20,000 ungated cells.

### Coupled cell cycle and cell proliferation assay

A 5'-bromo-2'-deoxyuridine (BrdU) flow kit (BD Pharmingen, San Diego, CA) was used to determine the cell cycle kinetics and to measure the incorporation of BrdU into DNA of proliferating cells. The assay was performed according to the manufacturer's protocol. Briefly, SBC5 cells (2 × 10^5 ^per well) were seeded overnight in 6-well tissue culture plates and treated with an optimized concentration of siRNAs in medium containing 10% FBS for 72 hours, followed by addition of 10 μM BrdU, and incubations continued for an additional 30 min. Both floating and adherent cells were pooled from triplicates wells per treatment point, fixed in a solution containing paraformaldehyde and the detergent saponin, and incubated for 1 hour with DNase at 37°C (30 μg per sample). FITC-conjugated anti-BrdU antibody (1:50 dilution in Wash buffer; BD Pharmingen, San Diego, CA) was added and incubation continued for 20 minutes at room temperature. Cells were washed in Wash buffer and total DNA was stained with 7-amino-actinomycin D (7-AAD; 20 μL per sample), followed by flow cytometric analysis using FACScan (BECKMAN COULTER) and total DNA content (7-AAD) was determined CXP Analysis Software Ver. 2.2 (BECKMAN COULTER).

### Microarray hybridization and statistical analysis for the clarification of down-stream genes

Purified total RNA was labeled and hybridized onto Affymetrix GeneChip U133 Plus 2.0 oligonucleotide arrays (Affymetrix, Santa Clara, CA) according to the manufacturer's instructions. Probe signal intensities were normalized by RMA and Quantile normalization methods (using R and Bioconductor). Next, signal intensity fluctuation due to inter-experimental variation was estimated. Each experiment was replicated (1 and 2), and the standard deviation (stdev) of log_2_(intensity_2_/intensity_1_) was calculated for each of a set of intensity ranges with the midpoints being at log_2_((intensity_1_+intensity_2_)/2) = 5, 7, 9, 11, 13, and 15. We modeled intensity variation using the formula stdev(log_2_(intensity_2_/intensity_1_)) = a * (log_2_((intensity_1_+intensity_2_)/2)) + *b *and estimated parameters *a *and *b *using the method of least squares. Using these values, the standard deviation of intensity fluctuation was calculated. The signal intensities of each probe were then compared between siKDM5B (EXP) and controls (siEGFP/siFFLuc) (CONT) and tested for up/down-regulation by calculating the *z*-score: log_2_(intensity_EXP_/intensity_CONT_)/(a * (log_2_((intensity_EXP_+intensity_CONT_)/2)) + *b*). Resultant *P*-values for the replication sets were multiplied to calculate the final *P*-value of each probe. These procedures were applied to each comparison: siEGFP vs. siKDM5B, siFFLuc vs. siKDM5B, and siEGFP vs. siFFLuc, respectively. We determined up/down-regulated gene sets as those that simultaneously satisfied the following criteria: (1) The Benjamini-Hochberg false discovery rate (FDR) < = 0.05 for siEGFP vs. siKDM5B, (2) FDR < = 0.05 for siFFLuc vs. siKDM5B and the regulation direction is the same as (1), and (3) siEGFP vs. siFFLuc has the direction opposite to (1) and (2) or *P *> 0.05 for siEGFP vs. siFFLuc. Finally, we performed a pathway analysis using the hyper-geometric distribution test, which calculates the probability of overlap between the up/down-regulated gene set and each GO category compared against another gene list that is randomly sampled. We applied the test to the identified up/down-regulated genes to test whether or not they are significantly enriched (FDR < = 0.05) in each category of "Biological processes" (857 categories) as defined by the Gene Ontology database.

### E2F reporter assay

We analyzed the transcriptional activity of E2F by the Cignal™ E2F Reporter Assay Kit (SuperArray Bioscience Corporation). A549 and SBC5 cells were treated with siRNAs (siEGFP and siKDM5B) and cultured for 24 hours. siRNA-treated cells were transfected with an E2F-responsive luciferase construct, which encodes the firefly *luciferase *reporter gene under the control of a minimal (m)CMV promoter and tandem repeats of the E2F transcriptional response element [TRE], negative control or positive control. After 24 hours of transfection, dual luciferase assay was performed using Dual-Luciferase Reporter Assay System (Promega), and promoter activity values are expressed as arbitrary units using a Renilla reporter for internal normalization.

Experiments were done in triplicate, and Student's *t*-test was used for statistical analysis.

## Competing interests

The authors declare that they have no competing interests.

## Authors' contributions

SH and RH designed this study and performed all experiments with the help of MY, MU and YI in Japan. DEN and JDK kindly provided patient samples and gave good advice. TT performed the statistical analysis of microarray. JDK, HIF, DEN, HY, BAJP and YN critically read the manuscript and gave good advice. SH and RH wrote this manuscript. All authors read and approved the final manuscript.

## Supplementary Material

Additional file 1 **Clinicopathologic characteristics and *KDM5B *expression**. Clinicopathologic information of bladder tumor tissues and *KDM5B *expression analyzed by quantitative real-time PCR. Click here for file

Additional file 2 **Clinicopathologic characteristics of bladder tissues on the tissue microarray**. Clinicopathologic information of bladder tumor tissues and KDM5B expression at the protein level. Click here for file

Additional file 3 **Clinicopathologic characteristics of lung tissues on the tissue microarray**. Clinicopathologic information of lung tumor tissues and KDM5B expression at the protein level. Click here for file

Additional file 4 **Expression of *KDM5B *in cancer tissues analyzed by cDNA microarray**. Elevated *KDM5B *expression in AML, breast cancer, CML, cervical cancer and renal cell carcinoma as well as bladder cancer and lung cancer in Japanese populations. Click here for file

Additional file 5 **Elevated *KDM5B *expression in various types of cancer**. Elevated *KDM5B *expression in AML, breast cancer, CML, cervical cancer and renal cell carcinoma in Japanese populations. Expression levels of *KDM5B *were compared between normal and tumor tissues. Signal intensity of each sample was analyzed by cDNA microarray, and the result is shown by box-whisker plot (median 50% boxed). Mann-Whitney's *U-*test was used for statistical analysis. Click here for file

Additional file 6 **Expression of *KDM5B *in cancer cell lines and FACS analysis stained with BrdU and 7-AAD after siKDM5B treatment**. (A) Expression of *KDM5B *in 12 bladder cancer cell lines, in four non-small cell lung cancer cell lines and one small cell lung cancer cell line. (B) Effect of siKDM5B on cell cycle kinetics in SBC5 cells. Cell cycle distribution was analyzed by flow cytometry after coupled staining with fluorescein isothiocyanate (FITC)-conjugated anti-BrdU and 7-amino-actinomycin D (7-AAD). Click here for file

Additional file 7 **Gene Ontology pathway analysis based on the Affymetrix's microarray data**. Gene Ontology pathway analysis was performed to clarify KDM5B functions in cancer cells. Click here for file

Additional file 8 **Two-dimensional, unsupervised hierarchical cluster analysis of SW780 and A549 mRNA expression profiles after knockdown of *KDM5B *expression**. Differentially expressed genes were selected for this analysis. Red, Up-regulated; Green, Down-regulated. Click here for file

Additional file 9 **Images of normal heart, kidney and liver stained by standard immunohistochemistry for protein expression of KDM5B**. We performed the control staining without primary antibody to eliminate the possibility of false-positive responses from the secondary antibody, and counterstaining was done with hematoxylin and eosin. Original magnification, ×40 and ×400. Click here for file

Additional file 10 **Subcellular localization of KDM5B in A549 cells**. A549 cells were subjected to cell cycle arrest by treatment with 7.5 μg/ml aphidicolin for 24 hours then immunocytochemically stained using anti-KDM5B monoclonal antibody (Alexa Fluor^® ^488 [green]), Phalloidin (F-actin, Alexa Fluor^® ^594 [red]) and 4',6'-diamidine-2'-phenylindole dihydrochloride (DAPI; [blue]) at 0, 4, 8, 12 and 24 hours after aphidicolin removal. Insets show FACS analysis demonstrating synchronized release of cell cycle arrest. A549 cells were fixed with PBS (-) containing 4% paraformaldehyde for 20 min, and rendered permeable with PBS (-) containing 0.1% Triton X-100 at room temperature for 2 min. Subsequently, the cells were covered with PBS (-) containing 3% bovine serum albumin for 1 hour at room temperature to block non-specific hybridization, and then were incubated with mouse anti-KDM5B antibody (1G10, Abnova), diluted at 1:100 ratio dilution. After washing with PBS (-), cells were stained by an Alexa Fluor^® ^488-conjugated anti-mouse secondary antibody (Molecular Probes, OR, USA) at 1:1000 dilution. Nuclei were counter-stained with 4',6'-diamidine-2'-phenylindole dihydrochloride (DAPI). Fluorescent images were obtained under a TCS SP2 AOBS microscope (Leica). Click here for file

Additional file 11 **Subcellular localization of KDM5B in SBC5 cells**. Same assay as Additional file 11, but using SBC5 cells. Click here for file

Additional file 12 **Primer sequences for quantitative RT-PCR**. Specific primer sequence for *GAPDH *(housekeeping gene), *SDH *(housekeeping gene), *KDM5B*, *E2F1 *and *E2F2*, respectively. Click here for file

Additional file 13 **siRNA sequences**. Sequences of siEGFP, siFFLuc, siNC (negative control), and si*KDM5B*, respectively. Click here for file
