# Supplementary material for: Overexpression of the JmjC histone demethylase KDM5B in human carcinogenesis: involvement in the proliferation of cancer cells through the E2F/RB pathway
Source: Mol Cancer. 2010 Mar 13;9:59. doi: 10.1186/1476-4598-9-59 (PMC2848192; doi:10.1186/1476-4598-9-59)
Supplement: Additional file 1 — Clinicopathologic characteristics and KDM5B expression. Clinicopathologic information of bladder tumor tissues and KDM5B expression analyzed by quantitative real-time PCR. [file 1476-4598-9-59-S1.PDF]

Additional file 1. Clinicopathologic characteristics and *KDM5B* expression

Tissue	Sample name	KDM5B expression	pT	Grade
Bladder tumor	BT2	3.723757677	T4	G3
	BT5	5.133204008	Ta	unknown
	BT6	4.188371931	Ta	G2
	BT8	2.392817477	Ta	G2
	BT9	4.383047299	Ta	G2
	BT10	5.688445406	T2	G3
	BT11	3.631539584	T2	G3
	BT12	3.378164362	T1	G2
	BT15	4.513809549	T2	G3
	BT16	3.307031343	Ta	G2
	BT18	3.225786495	Ta	G3
	BT20	7.264818254	T1	G2
	BT21	3.73186917	Ta	G3
	BT22	5.96353482	T2	G2
	BT23	4.124373502	T1	G2
	BT28	3.171562984	Ta	G1
	BT31	8.472334982	Ta	G2
	BT32	1.178595838	T2	G3
	BT33	2.01221958	T1	G2
	BT34	5.172175761	Ta	G2
	BT35	3.321182379	T3a	G3
	BT36	2.481706615	T2	G3
	BT38	4.937286373	Ta	G2
	BT39	5.87070444	T1	G3
	BT40	1.368281641	T2	G3
	BT41	1.14384635	T1	G2
	BT42	2.254269456	T2	G3
	BT43	5.80078388	Ta	G1
	BT44	8.624782522	T1	G2
	BT46	3.684335203	Ta	G2
	BT48	7.861600554	T2	G3
	BT49	4.282269434	Ta	G1
	BT50	8.482319486	T1	G3
	BT51	4.541882865	Ta	G2
	BT52	6.174337486	T3	G3
	BT53	7.942166599	Ta	G2
	BT54	1.688164538	T1	G3
	BT56	3.464832277	T2	G3
	BT57	4.616693966	T1	G2
	BT58	2.674320995	Ta	G2
	BT59	3.932299671	T2	G3
	BT60	8.114636683	Mets	G3
	BT64	8.550992233	Ta	G2
	BT66	4.093128255	Ta	G1
	BT67	5.058858835	T1	G2
	BT68	3.670499567	Ta	G2
	BT69	24.88108906	Ta	G2
	BT70	6.05734334	T1	G2
	BT71	4.133922672	T1	G3
	BT72	8.458194325	Ta	G1
	BT74	5.777832954	Ta	G1
	BT76	3.293270361	T1	G1
	BT77	4.147895826	Ta	G2
	BT78	2.834558288	T1	G3
	BT79	2.537326226	Ta	G2
	BT80	6.609713052	Ta	G2
	BT81	3.010341953	Ta	G2
	BT82	3.730894533	T1	G3
	BT83	8.337717858	Ta	G2
	BT84	10.6155221	Ta	G2
	BT85	3.972433124	T1	G2
	BT87	0.525080141	T2	G2
	BT88	10.60653495	T1	G3
	BT90	5.899961936	Ta	G2
	BT92	3.434195352	T1	G2
	BT93	2.468130141	T2	G3
	BT94	10.17964275	Ta	G1
	BT95	2.382499274	T3a	G3
	BT96	3.358390417	Ta	G1
	BT97	7.767841254	Ta	G2
	BT98	15.44824773	Ta	G2
	BT99	9.024065187	T1	G2
	BT100	7.666354252	T1	G3

Tissue	Sample name	KDM5B expression	pT	Grade
Bladder tumor	BT101	6.639631354	T2	G3
	BT103	8.359395135	T1	G2
	BT104	4.970020969	T4	G2
	BT105	4.013647709	T2	G2
	BT106	4.197667938	Ta	G3
	BT107	2.985594674	Mets	G3
	BT108	17.79609597	T1	G2
	BT109	4.178500086	Ta	G2
	BT110	3.897383041	T1	G2
	BT112	6.820473729	Ta	G3
	BT113	3.885112471	T1	G3
	BT114	2.031697472	T2	G3
	BT115	2.196992553	T1	G3
	BT116	5.575781632	T2a	G3
	BT119	6.928136024	Ta	G2
	BT120	10.88107536	Ta	G2
	BT122	4.566211985	T1	G3
	BT125	7.654271815	T1	G2
	BT127	7.136781851	T1	G2
	BT128	7.894791474	Ta	G1
	BT129	3.257744612	Ta	G2
	BT130	5.014176867	Ta	G2
	BT131	4.813191905	T2	G3
	BT132	3.100918885	T2	G3
	BT133	3.500649848	T1	G2
	BT135	3.160757145	T2	G3
	BT137	2.894619193	Ta	G2
	BT138	1.21990425	Ta	G1
	BT139	2.177775824	T2	G3
	BT140	5.729698929	Ta	G2
	BT141	2.063801268	Mets	G3
	BT143	2.82525806	T1	G3
	BT145	3.626263347	T2	G2
	BT150	0.96273313	Ta	G2
	BT151	3.69777386	Ta	G3
	BT152	2.168371128	Ta	G2
	BT154	7.815917508	T1	G3
	BT158	4.685174508	Ta	G2
	BT160	3.651623011	T1	G2
	BT161	7.75780893	Ta	G2
	BT162	1.139458522	T3	G3
	BT164	2.884535653	Ta	G1
	BT165	2.164550933	T2	G3
	BT169	0.965883667	T2	G3
	BT178	2.990188871	Ta	G2
	BT180	2.962694209	T1	G2
	BT181	3.508136742	T2	G3
	BT187	8.076148665	Ta	G2
	BT188	4.920557483	T2	G3
	BT189	4.396314262	T1	G2
Normal bladder	BN11A	2.041077373		
	BN12A	1.37836977		
	BN13A	1.309847551		
	BN14A	1.503884371		
	BN14B	1.556433786		
	BN15A	2.120084104		
	BN17B	1.815168518		
	BN19A	1.774206371		
	BN1A	2.793568931		
	BN20B	1.875164786		
	BN21A	3.430175314		
	BN22A	1.133256753		
	BN22B	1.759057821		
	BN25A	2.119878568		
	BN26A	0.997744917		
	BN2A	2.367327592		
	BN2B	2.440497064		
	BN4A	2.441758365		
	BN4B	2.064467556		
	BN5B	1.694005395		
	BN6A	2.011887643		
	BN8A	1.644228733		
	BN9A	2.237320664		
