# Supplementary material for: Overexpression of the JmjC histone demethylase KDM5B in human carcinogenesis: involvement in the proliferation of cancer cells through the E2F/RB pathway
Source: Mol Cancer. 2010 Mar 13;9:59. doi: 10.1186/1476-4598-9-59 (PMC2848192; doi:10.1186/1476-4598-9-59)
Supplement: Additional file 2 — Clinicopathologic characteristics of bladder tissues on the tissue microarray. Clinicopathologic information of bladder tumor tissues and KDM5B expression at the protein level. [file 1476-4598-9-59-S2.PDF]

Additional file 2. Clinicopathologic charcteristics of bladder tissues on the tissue microarray

Case No.	Age	Gender	Pathological diagnosis	Grade	TNM	KDM5B staining
1	71	Male	squamous cell carcinoma	G1	T1N0M0	++
2	60	Male	squamous cell carcinoma	G1	T2N0M0	+
3	76	Male	adenocarcinoma	G2	T2N0M0	++
4	50	Male	adenocarcinoma	G2	T2N0M0	++
5	68	Male	adenocarcinoma	G3	T2N0M0	+
6	74	Female	adenocarcinoma	G3	T2N0M0	+
7	27	Male	transitional cell carcinoma	G1	TisN0M0	+
8	50	Male	transitional cell carcinoma	G1	T1N0M0	+
9	49	Female	transitional cell carcinoma	G1	T1N0M0	++
10	67	Male	transitional cell carcinoma	G1	T1N0M0	++
11	51	Female	transitional cell carcinoma	G1	T1N0M0	+
12	57	Male	transitional cell carcinoma	G1	T1N0M0	+
13	47	Male	transitional cell carcinoma	G2	T2N0M0	-
14	54	Male	transitional cell carcinoma	G2	T2N0M0	-
15	45	Male	transitional cell carcinoma	G2	T1N0M0	-
16	74	Male	transitional cell carcinoma	G2	T2N0M0	+
17	51	Male	transitional cell carcinoma	G2	T1N0M0	+
18	80	Male	transitional cell carcinoma	G2	T2N0M0	-
19	53	Female	transitional cell carcinoma	G2	T1N0M0	-
20	37	Male	transitional cell carcinoma	G2	T2N0M0	+
21	55	Male	transitional cell carcinoma	G2	T4N2MX	++
22	52	Male	transitional cell carcinoma	G2	T1N0M0	-
23	78	Male	transitional cell carcinoma	G3	T1N0M0	+
24	64	Male	transitional cell carcinoma	G3	T3N2M1	+
25	70	Male	transitional cell carcinoma	G3	T2N0M0	-
26	61	Male	transitional cell carcinoma	G3	T2N0M0	+
27	61	Male	transitional cell carcinoma	G3	T1N0M0	-
28	39	Female	transitional cell carcinoma	G3	T2N0M0	-
29	30	Male	sarcoma	-	T2N0M0	-

(-) not detected
(+) weak or moderate
(++) strong
