# Supplementary material for: Overexpression of the JmjC histone demethylase KDM5B in human carcinogenesis: involvement in the proliferation of cancer cells through the E2F/RB pathway
Source: Mol Cancer. 2010 Mar 13;9:59. doi: 10.1186/1476-4598-9-59 (PMC2848192; doi:10.1186/1476-4598-9-59)
Supplement: Additional file 3 — Clinicopathologic characteristics of lung tissues on the tissue microarray. Clinicopathologic information of lung tumor tissues and KDM5B expression at the protein level. [file 1476-4598-9-59-S3.PDF]

Additional file 3. Clinicopathologic charcteristics of lung tissues on the tissue microarray

Case No.	Age	Gender	Pathological diagnosis	Tumor history	Tumor size (cm)	Differentiation	TNM	KDM5B staining
1	60	Male	lung metastasis (renal cell carcinoma)	3 M	3x3 x2.5	moderately	T2NxM1	++
2	N/A	N/A	adenocarcinoma	1 M	-	-	T0NxMx	+
3	N/A	N/A	squamous cell carcinoma	1 M	-	-	T0NxMx	-
4	60	Male	squamous cell carcinoma	1 M	5x4.5x4	poorly	T2N0M0	++
5	47	Female	adenocarcinoma	0.5 M	5x4x3.5	poorly	T2N0M0	-
6	53	Female	squamous cell carcinoma	0.5 M	N/A	moderately	T0N0M0	+
7	40	Male	squamous cell carcinoma	5 M	3.9x3.5x2.5	moderately	T2N0M0	++
8	56	Female	adenocarcinoma	3 M	3.5x3x3	poorly	T2N0M0	+
9	49	Male	squamous cell carcinoma	12 M	3.4x2.9x2.5	moderately	T2N0M0	+
10	45	Female	bronchio alveolar carcinoma	1 Y+	4x3x2	N/A	T2N0M0	-
11	34	Female	fibrosarcoma	1 M	N/A	moderately	T0N0M0	-
12	50	Male	bronchio alveolar carcinoma	2 M	9x6.5x5	N/A	T3N0M0	-
13	57	Male	squamous cell carcinoma	1 M	5x4x2.5	poorly	T2N0M0	++
14	65	Male	atypical carcinoma (central type)	2 M	8x7x5	moderately	T3N0M0	+
15	36	Female	adenocarcinoma, mucous	1 M	4x4x3.5	well	T2N0M0	++
16	57	Male	squamous cell carcinoma	2 M	4x3.5x5	moderately	T2N0M0	-
17	29	Male	squamous cell carcinoma	3 M	diameter 3.5	moderately	T2N0M0	+
18	52	Male	small cell carcinoma	10 D	4.5x4x3.5	poorly	T2N0M0	-
19	63	Male	squamous cell carcinoma (cornifying)	1 M+	7.5x5x3	moderately	T3N0M0	-
20	68	Male	adenocarcinoma, papillary (peripheral type)	1 M	diameter 3	well	T2N1M0	-
21	57	Male	squamous cell carcinoma (central type, cornifying)	5 M	3x2x2	well	T2N0M0	+
22	52	Male	squamous cell carcinoma	6 M	5.5x3x2.5	moderately	T2N0M0	+
23	46	Male	squamous cell carcinoma (cornifying)	1 M+	6x5x4	well	T3N0M0	-
24	58	Male	squamous cell carcinoma (central type)	3 M	3x2	moderately	T2N1M0	+
25	63	Male	adenocarcinoma	2 weeks+	6.5x6x1	moderately	T3N0M0	-
26	61	Female	bronchio alveolar carcinoma	4 M+	3.5x3.5x2	well	T2N0M0	+
27	40	Male	squamous cell carcinoma	2 M	6x4x2	well	T3N1M0	+
28	64	Male	squamous cell carcinoma	3 M	8x7x9	moderately	T3N0M0	+
29	44	Female	adenosquamous carcinoma	6 M	5x5x3.5	moderately	T2N1M0	-
30	61	Male	squamous cell carcinoma	5 M	4x4x3.5	well	T2N0M0	+
31	65	Female	squamous cell carcinoma	3 M+	diameter 2.5	poorly	T1N0M0	-
32	64	Female	adenocarcinoma, papillary (peripheral type)	1 M	4x4.5	well	T2N0M0	-
33	70	Male	adenosquamous carcinoma	N/A	diameter 4	moderately	T2N1M0	-
34	68	Male	small cell carcinoma	1 M	3.7x3x2	poorly	T2N0M0	+
35	65	Male	carcinoma (peripheral type)	1 M	4x4x3	moderately	T2N0M0	++
36	59	Female	adenocarcinoma, papillary	1 M+	4x3.5x2.5	well	T2N0M0	++
37	67	Male	squamous cell carcinoma	2 M+	diameter 6	moderately	T2N0M0	+
38	70	Male	squamous cell carcinoma	1 M+	diameter 3.2	poorly	T2N0M0	++
39	47	Female	adenocarcinoma	14 D+	4.5x4x3.5	moderately	T2N0M0	-
40	71	Male	squamous cell carcinoma	5 M	4x2.5	moderately	T2N0M0	+
41	65	Male	squamous cell carcinoma	2 M+	15x10x12	moderately	T2N0M0	++
42	68	Male	adenocarcinoma, squamous cell carcinoma	1 M	8x4x4	moderately	T3N0M0	++
43	39	Female	adenocarcinoma	1 M	diameter 6	moderately	T2N1M0	+
44	67	Male	squamous cell carcinoma	18 M	5x3.5x2.5	moderately	T2N1M0	+
45	60	Female	alveolus cell carcinoma	1 M	3x2.5x2	N/A	T2N0M0	++
46	70	Female	carcinoma	1 M	diameter 2	moderately	T1N0M0	+
47	27	Male	lung metastasis (sarcoma)	5 Y	4x3.5x3	moderately	T2NxM1	-
48	65	Male	squamous cell carcinoma	2 M+	15x10x12	moderately	T3N0M0	-
49	68	Female	squamous cell carcinoma	4 M	3x5	moderately	T2N0M0	+
50	58	Female	adenocarcinoma	3 M	3.7x3x2	moderately	T2N1M0	++
51	68	Male	squamous cell carcinoma	1 M+	5x5x3	well	T2N0M0	+
52	48	Male	squamous cell carcinoma	5 M	7x6x4	moderately	T3N0M0	+
53	59	Male	squamous cell carcinoma	2 Y	diameter 2	N/A	T1N0M0	+
54	54	Male	adenocarcinoma, cyst	2 M	5.5x3x3	moderately	T2N1M0	+
55	45	Male	squamous cell carcinoma	5 D	6.5x4.5x3	moderately	T3N0M0	++
56	69	Male	squamous cell carcinoma	40 D	5x4x4	poorly	T2N1M0	++
57	78	Female	alveolus cell carcinoma	2 M+	2.4x2x1.7	moderately	T1N0M0	+
58	60	Male	adenocarcinoma	1 M+	2x2x1	moderately	T1N0M0	+
59	54	Female	alveolus cell carcinoma	1 M+	4x2.5x2	moderately	T2N1M0	+
60	78	Male	alveolus cell carcinoma	6 M+	1.5x0.5x0.3	moderately	T1N0M0	++
61	70	Male	alveolus cell carcinoma	1 week	2x1.7x0.8	well	T1N0M0	+
62	45	Female	bronchio alveolar carcinoma	1 M+	diameter 6	moderately	T2N0M0	++

(-) not detected
(+) weak or moderat
(++) strong
