# Supplementary material for: Overexpression of the JmjC histone demethylase KDM5B in human carcinogenesis: involvement in the proliferation of cancer cells through the E2F/RB pathway
Source: Mol Cancer. 2010 Mar 13;9:59. doi: 10.1186/1476-4598-9-59 (PMC2848192; doi:10.1186/1476-4598-9-59)
Supplement: Additional file 4 — Expression of KDM5B in cancer tissues analyzed by cDNA microarray. Elevated KDM5B expression in AML, breast cancer, CML, cervical cancer and renal cell carcinoma as well as bladder cancer and lung cancer in Japanese populations. [file 1476-4598-9-59-S4.PDF]

Additional file 4. Expression of *KDM5B* in cancer tissues analyzed by cDNA microarray*

		Ratio (Tumor / Normal)			
**Tissue type**	**Case (n)**	**>2 fold**	**>3 fold**	**>5 fold**	**>10 fold**
Acute myelogenous leukemia (AML)	56	43 (76.8%)	39 (69.6%)	22 (39.3%)	0 (0%)
Bladder cancer	34	21 (61.8%)	6 (17.6%)	3 (8.8%)	0 (0%)
Breast cancer	80	33 (41.3%)	11 (13.8%)	3 (3.8%)	0 (0%)
Cervical cancer	19	16 (84.2%)	13 (68.4%)	8 (42.1%)	1 (5.3%)
Chronic myelogenous leukemia (CML)	74	45 (60.8%)	24 (32.4%)	6 (8.1%)	2 (2.7%)
Non-small cell lung cancer (NSCLC)	37	28 (75.7%)	18 (48.6%)	7 (18.9%)	0 (0%)
Renal cell carcinoma (RCC)	24	10 (41.7%)	1 (4.2%)	0 (0%)	0 (0%)
Small cell lung cancer (SCLC)	15	11 (73.3%)	7 (46.7%)	6 (40%)	0 (0%)

***** We compared the signal intensity of *KDM5B* between tumor and corresponding non-neoplastic tissues derived from the same patient.
