# Supplementary figures and images for: Overexpression of the JmjC histone demethylase KDM5B in human carcinogenesis: involvement in the proliferation of cancer cells through the E2F/RB pathway
Source: Mol Cancer. 2010 Mar 13;9:59. doi: 10.1186/1476-4598-9-59 (PMC2848192; doi:10.1186/1476-4598-9-59)

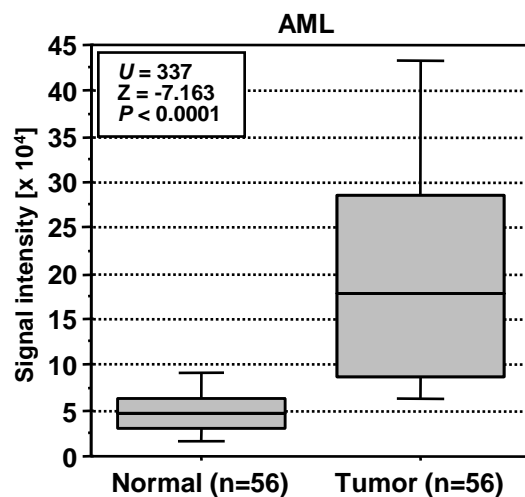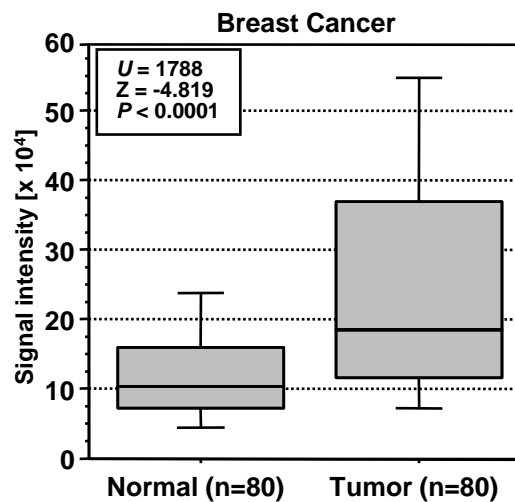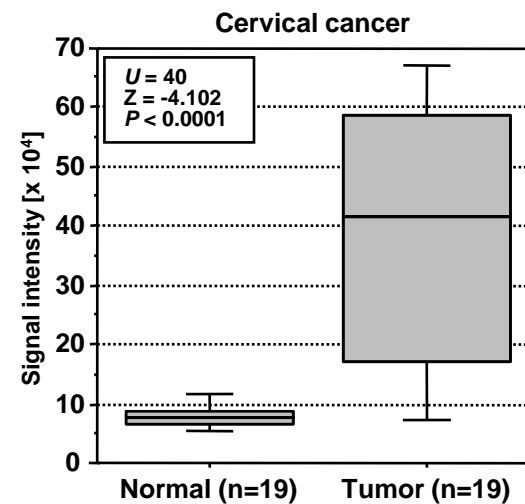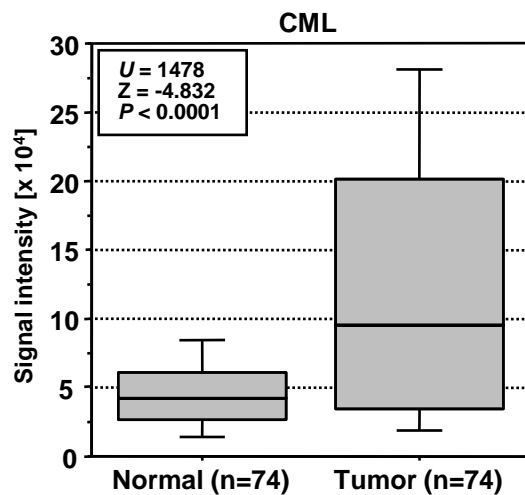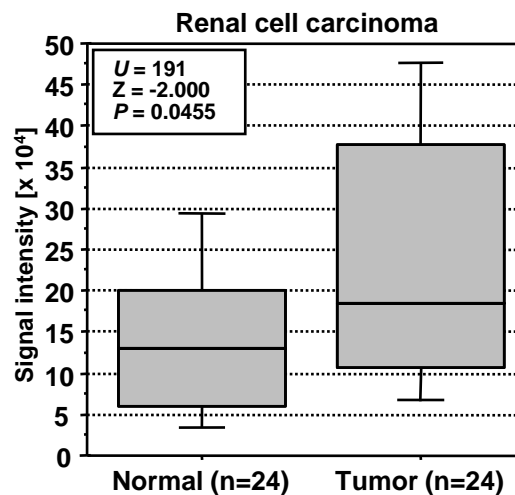

Supplement: Additional file 5 — Elevated KDM5B expression in various types of cancer. Elevated KDM5B expression in AML, breast cancer, CML, cervical cancer and renal cell carcinoma in Japanese populations. Expression levels of KDM5B were compared between normal and tumor tissues. Signal intensity of each sample was analyzed by cDNA microarray, and the result is shown by box-whisker plot (median 50% boxed). Mann-Whitney's U-test was used for statistical analysis. [file 1476-4598-9-59-S5.PDF]

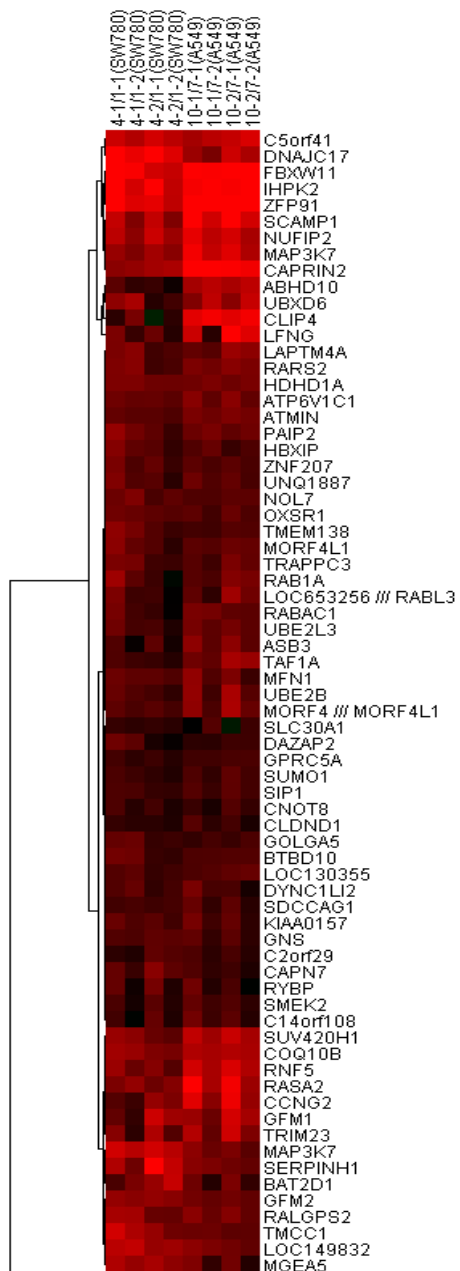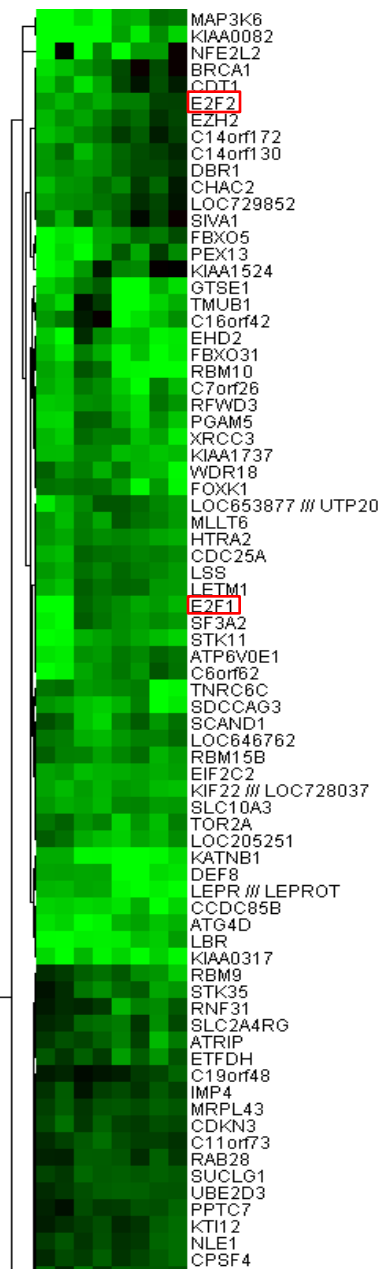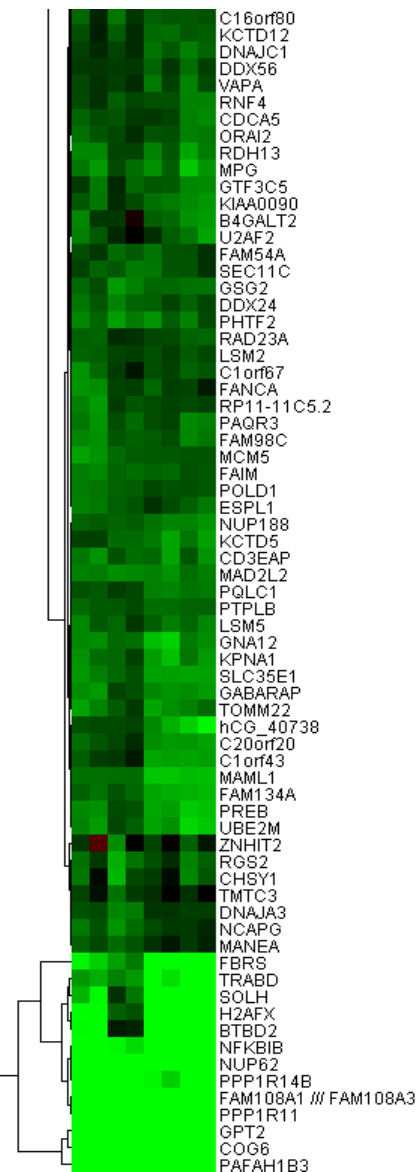

Supplement: Additional file 8 — Two-dimensional, unsupervised hierarchical cluster analysis of SW780 and A549 mRNA expression profiles after knockdown of KDM5B expression. Differentially expressed genes were selected for this analysis. Red, Up-regulated; Green, Down-regulated. [file 1476-4598-9-59-S8.PDF]

A549\_0hr

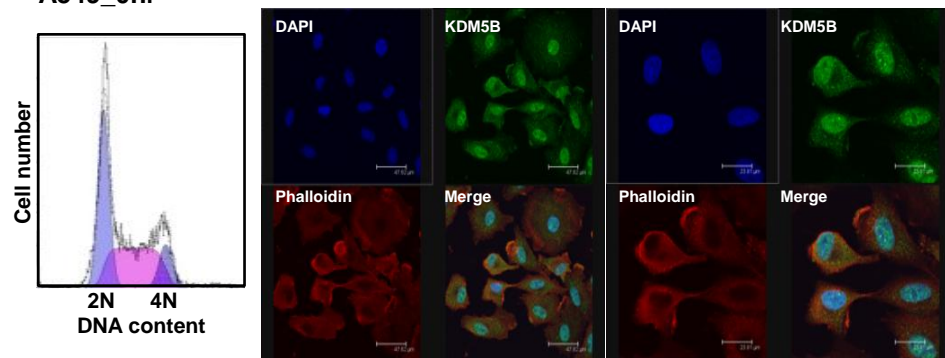

A549\_12hr

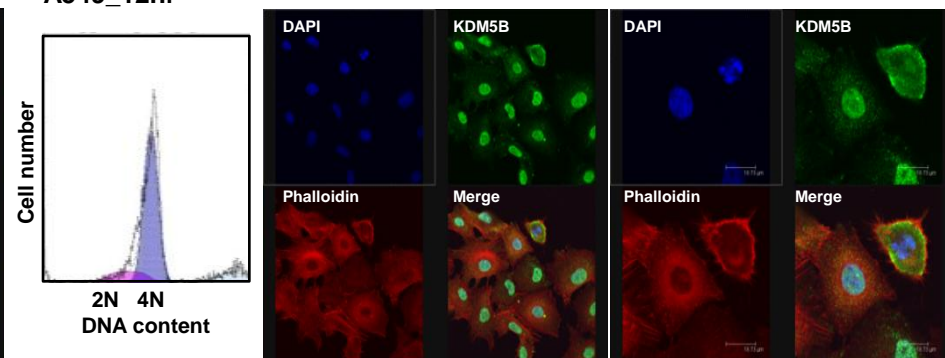

A549\_4hr

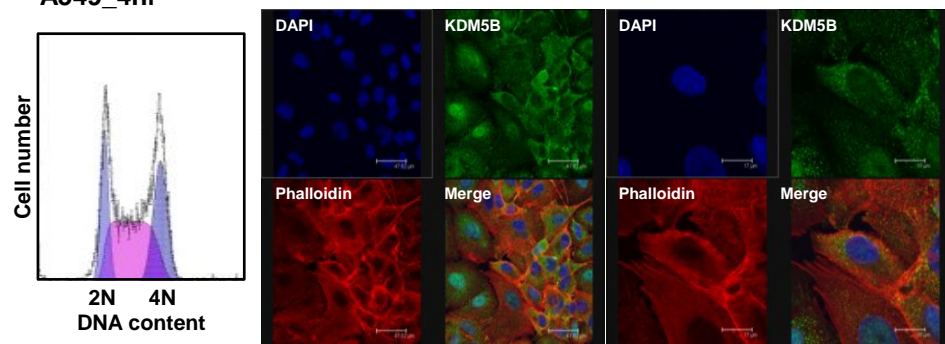

A549\_24hr

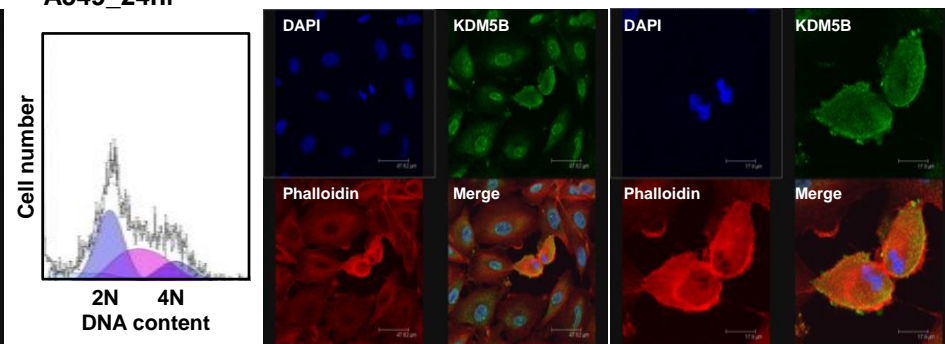

A549\_8hr

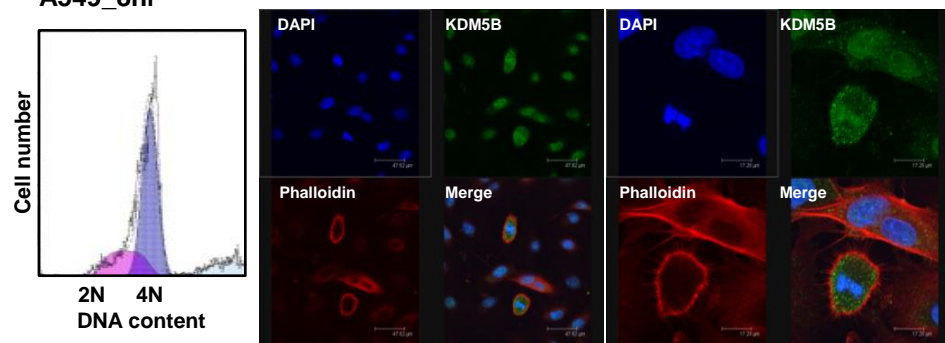

Supplement: Additional file 10 — Subcellular localization of KDM5B in A549 cells. A549 cells were subjected to cell cycle arrest by treatment with 7.5 μg/ml aphidicolin for 24 hours then immunocytochemically stained using anti-KDM5B monoclonal antibody (Alexa Fluor® 488 [green]), Phalloidin (F-actin, Alexa Fluor® 594 [red]) and 4',6'-diamidine-2'-phenylindole dihydrochloride (DAPI; [blue]) at 0, 4, 8, 12 and 24 hours after aphidicolin removal. Insets show FACS analysis demonstrating synchronized release of cell cycle arrest. A549 cells were fixed with PBS (-) containing 4% paraformaldehyde for 20 min, and rendered permeable with PBS (-) containing 0.1% Triton X-100 at room temperature for 2 min. Subsequently, the cells were covered with PBS (-) containing 3% bovine serum albumin for 1 hour at room temperature to block non-specific hybridization, and then were incubated with mouse anti-KDM5B antibody (1G10, Abnova), diluted at 1:100 ratio dilution. After washing with PBS (-), cells were stained by an Alexa Fluor® 488-conjugated anti-mouse secondary antibody (Molecular Probes, OR, USA) at 1:1000 dilution. Nuclei were counter-stained with 4',6'-diamidine-2'-phenylindole dihydrochloride (DAPI). Fluorescent images were obtained under a TCS SP2 AOBS microscope (Leica). [file 1476-4598-9-59-S10.PDF]

SBC5\_0hr

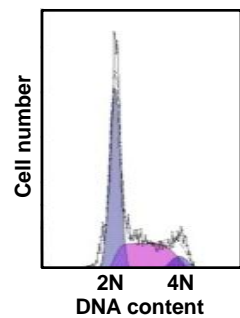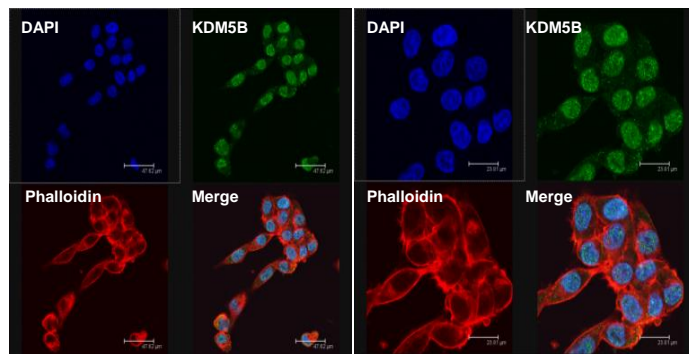

SBC5\_12hr

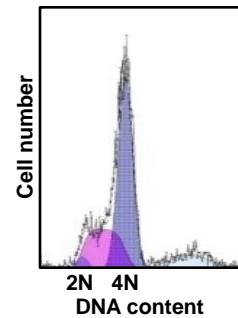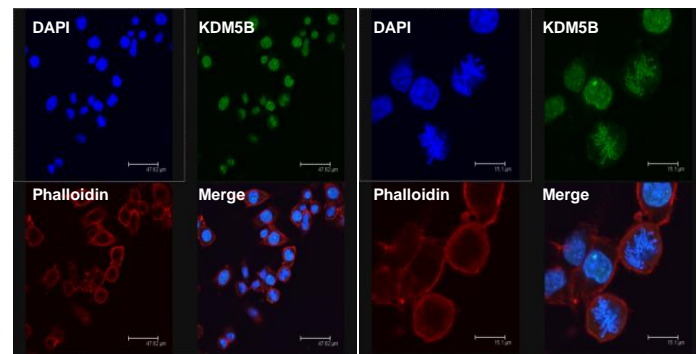

SBC5\_4hr

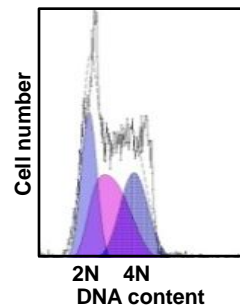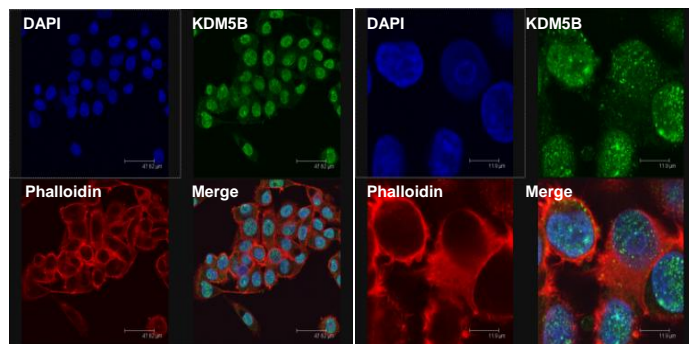

SBC5\_24hr

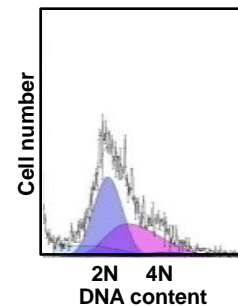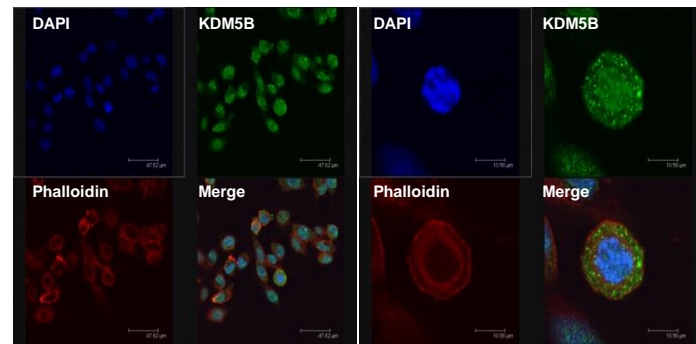

SBC5\_8hr

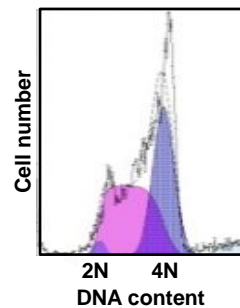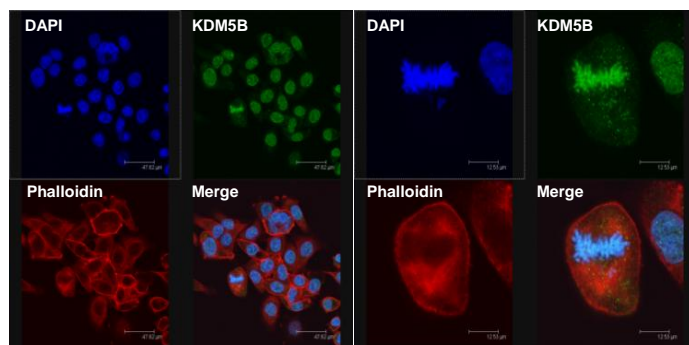

Supplement: Additional file 11 — Subcellular localization of KDM5B in SBC5 cells. Same assay as Additional file 11, but using SBC5 cells. [file 1476-4598-9-59-S11.PDF]
