# Supplementary material for: Overexpression of the JmjC histone demethylase KDM5B in human carcinogenesis: involvement in the proliferation of cancer cells through the E2F/RB pathway
Source: Mol Cancer. 2010 Mar 13;9:59. doi: 10.1186/1476-4598-9-59 (PMC2848192; doi:10.1186/1476-4598-9-59)
Supplement: Additional file 6 — Expression of KDM5B in cancer cell lines and FACS analysis stained with BrdU and 7-AAD after siKDM5B treatment. (A) Expression of KDM5B in 12 bladder cancer cell lines, in four non-small cell lung cancer cell lines and one small cell lung cancer cell line. (B) Effect of siKDM5B on cell cycle kinetics in SBC5 cells. Cell cycle distribution was analyzed by flow cytometry after coupled staining with fluorescein isothiocyanate (FITC)-conjugated anti-BrdU and 7-amino-actinomycin D (7-AAD). [file 1476-4598-9-59-S6.PDF]

**A**

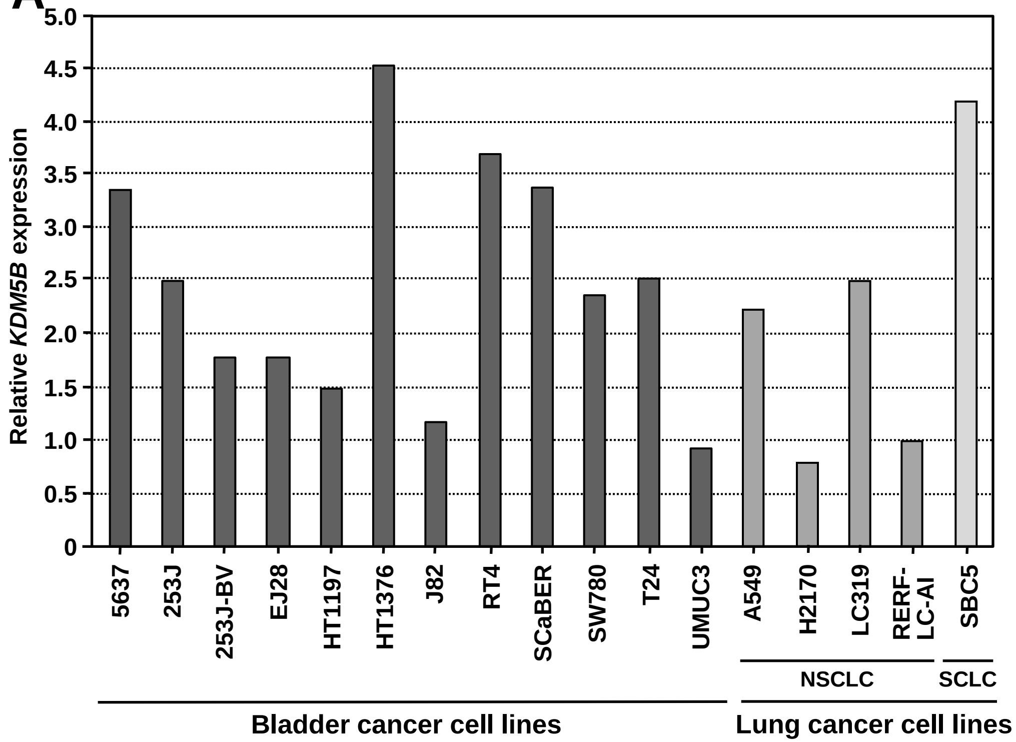


**B**

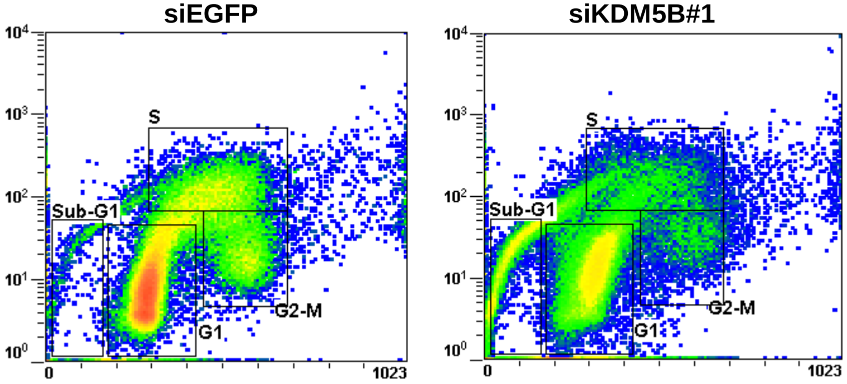


**The proportion of cells in each cell cycle phase**

| siRNA | Phase (%) Sub-$G_1$ | $G_1$ | S | $G_2$/M |
|---|---|---|---|---|
| siEGFP | 1.00 | 71.63 | 15.42 | 11.94 |
| siKDM5B#1 | 18.71 | 62.30 | 12.75 | 6.30 |
