# Supplementary material for: Overexpression of the JmjC histone demethylase KDM5B in human carcinogenesis: involvement in the proliferation of cancer cells through the E2F/RB pathway
Source: Mol Cancer. 2010 Mar 13;9:59. doi: 10.1186/1476-4598-9-59 (PMC2848192; doi:10.1186/1476-4598-9-59)
Supplement: Additional file 7 — Gene Ontology pathway analysis based on the Affymetrix's microarray data. Gene Ontology pathway analysis was performed to clarify KDM5B functions in cancer cells. [file 1476-4598-9-59-S7.PDF]

Additional file 7. Gene Ontology pathway analysis based on the Affymetrix's microarray data

Entry ID	Name	Definition	*P*
GO:0007049	Cell cycle	The progression of biochemical and morphological phases and events that occur in a cell during successive cell replication or nuclear replication events.	1.15×10^{-9}
GO:0022403	Cell cycle phase	A cell cycle process comprising the steps by which a cell progresses through one of the biochemical and morphological phases and events that occur during successive cell replication or nuclear replication events.	2.12×10^{-8}
GO:0022402	Cell cycle process	A cellular process that is involved in the progression of biochemical and morphological phases and events that occur in a cell during successive cell replication or nuclear replication events.	8.26×10^{-8}
GO:0000278	Mitotic cell cycle	Progression through the phases of the mitotic cell cycle, the most common eukaryotic cell cycle, which canonically comprises four successive phases called G1, S, G2, and M and includes replication of the genome and the subsequent segregation of chromosomes into daughter cells. In some variant cell cycles nuclear replication or nuclear division may not be followed by cell division, or G1 and G2 phases may be absent.	2.80×10^{-7}
GO:0000279	M phase	Progression through M phase, the part of the cell cycle comprising nuclear division.	1.45×10^{-6}
GO:0007067	Mitosis	Progression through mitosis, the division of the eukaryotic cell nucleus to produce two daughter nuclei that, usually, contain the identical chromosome complement to their mother.	2.41×10^{-6}
GO:0000087	M-phase of mitotic cell cycle	Progression through M phase, the part of the mitotic cell cycle during which mitosis takes place.	2.96×10^{-6}
GO:0043283	Biopolymer metabolic process	The chemical reactions and pathways involving biopolymers, long, repeating chains of monomers found in nature, such as polysaccharides and proteins.	1.91×10^{-5}
GO:0044237	Cellular metabolic process	The chemical reactions and pathways by which individual cells transform chemical substances.	5.12×10^{-5}
GO:0044238	Primary metabolic process	The chemical reactions and pathways involving those compounds which are formed as a part of the normal anabolic and catabolic processes. These processes take place in most, if not all, cells of the organism.	5.44×10^{-5}
GO:0043170	Macromolecule metabolic process	The chemical reactions and pathways involving those compounds which are formed as a part of the normal anabolic and catabolic processes. These processes take place in most, if not all, cells of the organism.	5.76×10^{-5}
GO:0000084	S phase of mitotic cell cycle	Progression through S phase, the part of the mitotic cell cycle during which DNA synthesis takes place.	1.38×10^{-4}
GO:0051320	S phase	Progression through S phase, the part of the mitotic cell cycle during which DNA synthesis takes place.	1.38×10^{-4}
GO:0051320	Cell division	The process resulting in the physical partitioning and separation of a cell into daughter cells.	1.47×10^{-4}
GO:0006996	Organelle organization and biogenesis	A process that is carried out at the cellular level which results in the formation, arrangement of constituent parts, or disassembly of an organelle within a cell. An organelle is an organized structure of distinctive morphology and function. Includes the nucleus, mitochondria, plastids, vacuoles, vesicles, ribosomes and the cytoskeleton. Excludes the plasma membrane.	1.97×10^{-4}
GO:0006260	DNA replication	The process whereby new strands of DNA are synthesized. The template for replication can either be an existing DNA molecule or RNA.	2.38×10^{-4}
