# Supplementary material for: Overexpression of the JmjC histone demethylase KDM5B in human carcinogenesis: involvement in the proliferation of cancer cells through the E2F/RB pathway
Source: Mol Cancer. 2010 Mar 13;9:59. doi: 10.1186/1476-4598-9-59 (PMC2848192; doi:10.1186/1476-4598-9-59)
Supplement: Additional file 9 — Images of normal heart, kidney and liver stained by standard immunohistochemistry for protein expression of KDM5B. We performed the control staining without primary antibody to eliminate the possibility of false-positive responses from the secondary antibody, and counterstaining was done with hematoxylin and eosin. Original magnification, ×40 and ×400. [file 1476-4598-9-59-S9.PDF]

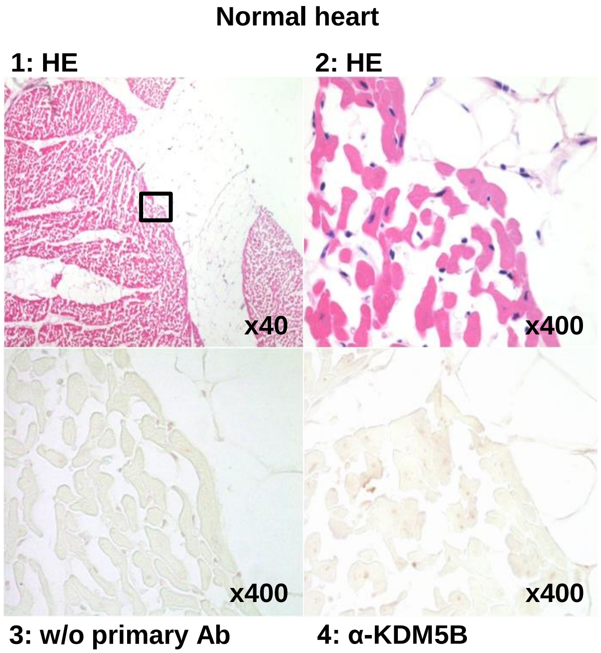
Normal heart
1: HE
2: HE
x40
x400
x400
x400
3: w/o primary Ab
4: α-KDM5B

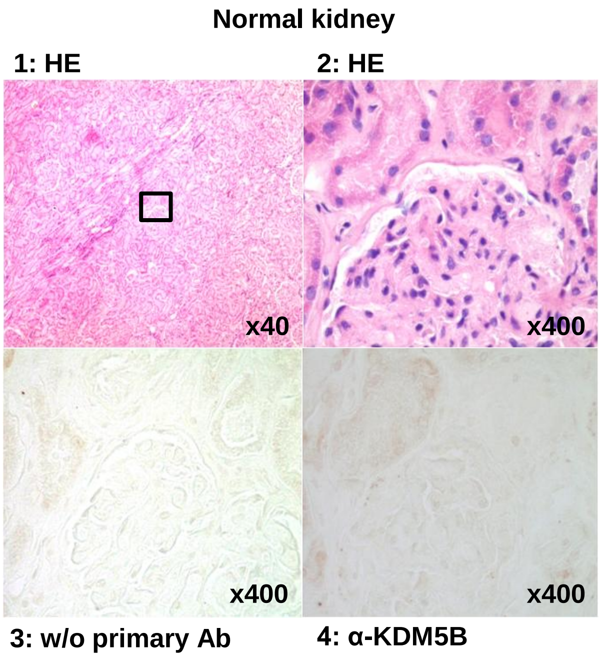
Normal kidney
1: HE
2: HE
x40
x400
x400
x400
3: w/o primary Ab
4: α-KDM5B

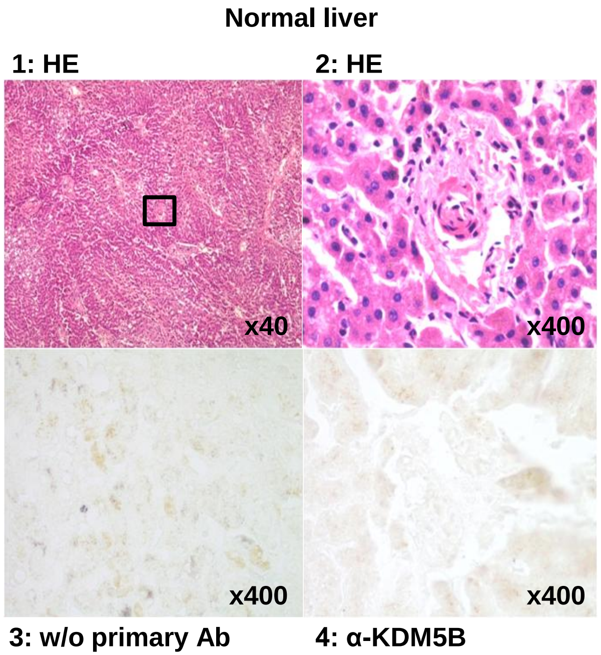
Normal liver
1: HE
2: HE
x40
x400
x400
x400
3: w/o primary Ab
4: α-KDM5B
