# Supplementary material for: Overexpression of the JmjC histone demethylase KDM5B in human carcinogenesis: involvement in the proliferation of cancer cells through the E2F/RB pathway
Source: Mol Cancer. 2010 Mar 13;9:59. doi: 10.1186/1476-4598-9-59 (PMC2848192; doi:10.1186/1476-4598-9-59)
Supplement: Additional file 12 — Primer sequences for quantitative RT-PCR. Specific primer sequence for GAPDH (housekeeping gene), SDH (housekeeping gene), KDM5B, E2F1 and E2F2, respectively. [file 1476-4598-9-59-S12.PDF]

Additional file 12. Primer sequences for quantitative RT-PCR

**Gene name**	**Primer sequence**
GAPDH (housekeeping gene) - f	5’ GCAAATTCCATGGCACCGTC 3’
GAPDH (housekeeping gene) - r	5’ TCGCCCCACTTGATTTTGG 3’
SDH (housekeeping gene) - f	5’ TGGGAACAAGAGGGCATCTG 3’
SDH (housekeeping gene) - r	5’ CCACCACTGCATCAAATTCATG 3’
KDM5B - f1	5’ ATTGCCTCAAAGGAATTTGGCAGTG 3’
KDM5B - r1	5’ CATCACTGGCATGTTGTTCAAATTC 3’
KDM5B - f2	5’ TGTCACAGTGGAATATGGAGCTGAC 3’
KDM5B - r2	5’ GCCACTATCAAGATACTCCTCTTCC 3’
E2F1 - f	5’ GCTGGACCACCTGATGAATATC 3’
E2F1 - r	5’ TCTGCAATGCTACGAAGGTCCTG 3’
E2F2 - f	5’ TGGCAACTTTAAGGAGCAGACAG 3’
E2F2 - r	5’ GGGCACAGGTAGACTTCGATGG 3’
