# Supplementary material for: Overexpression of the JmjC histone demethylase KDM5B in human carcinogenesis: involvement in the proliferation of cancer cells through the E2F/RB pathway
Source: Mol Cancer. 2010 Mar 13;9:59. doi: 10.1186/1476-4598-9-59 (PMC2848192; doi:10.1186/1476-4598-9-59)
Supplement: Additional file 13 — siRNA sequences. Sequences of siEGFP, siFFLuc, siNC (negative control), and siKDM5B, respectively. [file 1476-4598-9-59-S13.PDF]

Additional file 13. siRNA sequences

siRNA name		Sequence
siEGFP		Sense: 5’ GCAGCACGACUUCUUCAAGTT 3’<br>Antisense: 5’ CUUGAAGAAGUCGUGCUGCTT 3’
siFFLuc		Sense: 5’ GUGCGCUGCUGGUGCCAACTT 3’<br>Antisense: 5’ GUUGGCACCAGCAGCGCACTT 3’
siNegative control (Cocktail)	Target#1	Sense: 5’ AUCCGCGCGAUAGUACGUA 3’<br>Antisense: 5’ UACGUACUAUCGCGCGGAU 3’
	Target#2	Sense: 5’ UUACGCGUAGCGUAAUACG 3’<br>Antisense: 5’ CGUAUUACGCUACGCGUAA 3’
	Target#3	Sense: 5’ UAUUCGCGCGUAUAGCGGU 3’<br>Antisense: 5’ ACCGCUAUACGCGCGAAUA 3’
siKDM5B#1		Sense: 5’ CAGUGAAUGAGCUCCGGCATT 3’<br>Antisense: 5’ UGCCGGAGCUCAUUCACUGTT 3’
siKDM5B#2		Sense: 5’ GGAAUAUGGAGCUGACAUUTT 3’<br>Antisense: 5’ AAUGUCAGCUCCAUAUUCCTT 3’
